# Deciphering the Odorant Binding, Activation, and Discrimination Mechanism of Dhelobp21 from *Dastarus Helophoroides*

**DOI:** 10.1038/s41598-018-31959-5

**Published:** 2018-09-10

**Authors:** Guang-Qiang Yu, Dong-Zhen Li, Yu-Lin Lu, Ya-Qi Wang, De-Xin Kong, Man-Qun Wang

**Affiliations:** 1 Hubei Insect Resources Utilization and Sustainable Pest Management Key Laboratory, College of Plant Science and Technology, Huazhong Agricultural University, Wuhan, 430070 P. R. China; 2 Agricultural Bioinformatics Key Laboratory of Hubei Province, College of Informatics, Huazhong Agricultural University, Wuhan, 430070 P. R. China

## Abstract

Odorant-binding proteins (OBPs) play a pivotal role in transporting odorants through the sensillar lymph of insect chemosensory sensilla and increasing the sensitivity of the olfactory system. To address the ligand binding, activation, and release mechanisms of OBPs, we performed a set of conventional molecular dynamics simulations for binding of the odorant-binding protein DhelOBP21 from *Dastarcus helophoroides* with 18 ligands (1-NPN and 17 volatiles), as well as four constant-pH molecular dynamics simulations. We found that the open pocket DhelOBP21 at pH 5.0 could bind volatiles and form a closed pocket complex via transformation of its N-terminus into regular Helix at pH 7.0 and vice versa. Moreover, the discrimination of volatiles (selectivity and promiscuity) was determined by the characteristics of both the volatiles and the ‘essential’ and ‘selective’ amino acid residues in OBP binding pockets, rather than the binding affinity of the volatiles. This study put forward a new hypothesis that during the binding of volatiles there are two transitions for the DhelOBP21 amino-terminus: pH- and odorant binding-dependent random-coil-to-helix. Another important finding is providing a framework for the exploration of the complete coil-to-helix transition process and theoretically analyzing its underlying causes at molecular level.

## Introduction

Olfaction is vital for organisms, and involved in a number of behaviors such as feeding, reproduction, and predator avoidance^1^. When odorants enter the aqueous sensillar lymph of the insect antennae, they are recognized, bound, and solubilized by odorant binding proteins (OBPs, also known as pheromone binding proteins, PBPs)^2^, which shuttle the odorants to olfactory receptors to evoke signal transduction^3,4^. OBPs are a family of small, soluble proteins which are highly expressed in sensillar lymph^5^. They have a hydrophobic binding cavity and can bind ligands with 10 to 20 carbon atoms^6^. According to the number of conserved cysteine residues, they are classified into different subfamilies: classical OBPs with six conserved cysteines, Minus-C OBPs with four cysteines, and Plus-C OBPs with more than six cysteines^7–9^.

**Figure 1:**
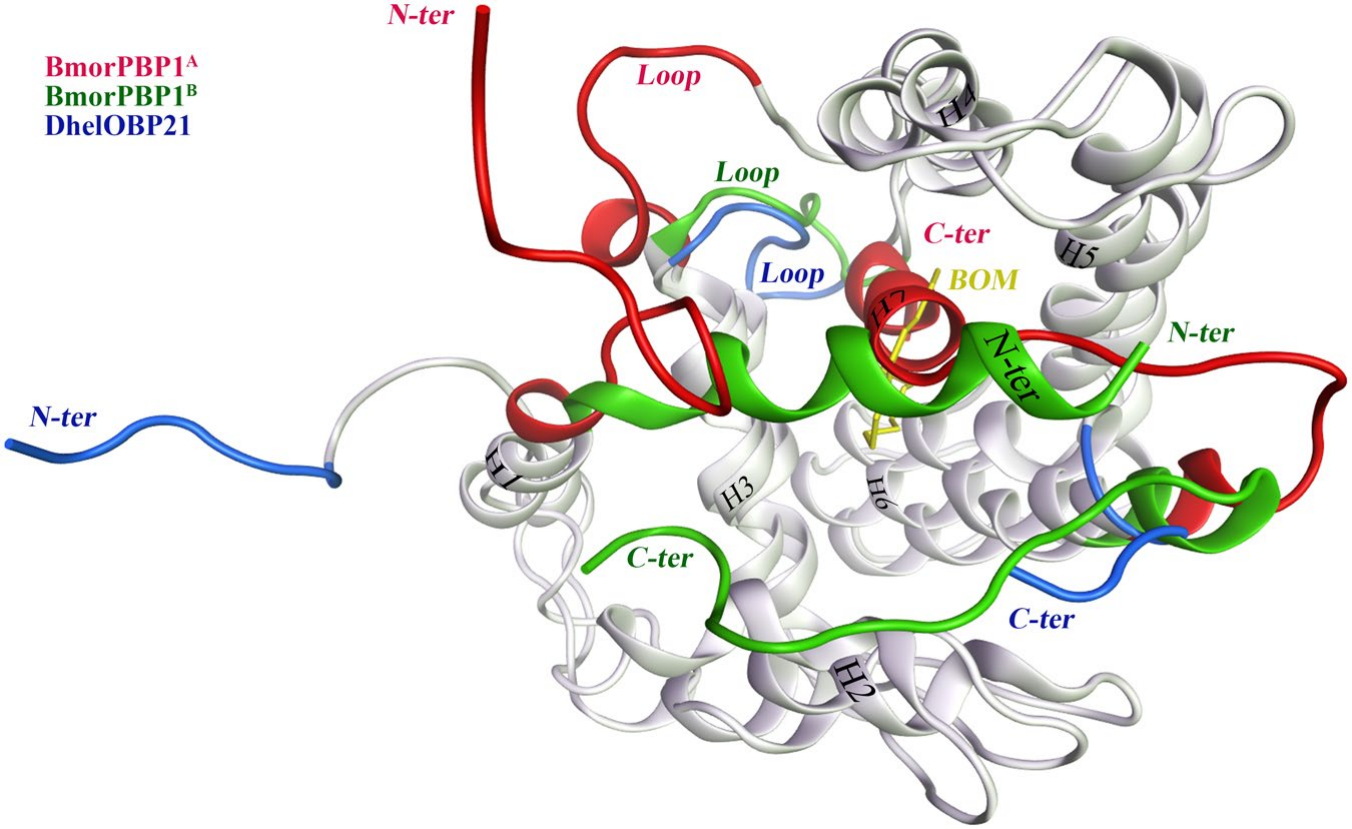
The superimposed structures of the BmorPBP1^A^ (red), BmorPBP1^B^ (green) and DhelOBP21 (blue). Remarkable differences were located in the N- and C- termini and the loop between helix α_3_ and α_4_ (residues 60 to 68, red, green and blue, respectively). The ligand BOM (Bombykol, in yellow) belonged to the BmorPBP1^B^.

Nevertheless, how OBPs capture, differentiate, transfer, and release odorants is controversial. The BmorPBP1, a classical OBP from *Bombyx mori*, has been well studied for the last two decades. According to the known crystal structures^9–15^ and biological assay studies^16–19^, odorant binding and release by BmorPBP1 is highly pH-dependent^14,18^ (Fig. 1). BmorPBP1 binds odorants at physiological pH (~7.0) and then releases them at a lower pH (~4.5)^10,16,20^ as a result of a conformational transition from BmorPBP1^B^ to BmorPBP1^A^. In the compacted structure of *holo* BmorPBP1^B^ (PDB code: 1DQE), a carboxyl (C)-terminal dodecapeptide segment resides near the binding pocket as an elongated loop/coil, alongside helix α_1_ in the amino (N)-terminus. By contrast, in *apo* BmorPBP1^A^ (PDB code: 1GM0)^12^, the C-terminus loop/coil forms a new helix, α_7_, which occupies the site where the ligand ought to bind, leading to the dissociation of the odorant from the pocket of BmorPBP1. Two possible exit passages have been proposed: one is the ‘lid’, namely, the loop of residues 60–68, and the other consists of the N-terminal and C-terminal segments that are arranged in parallel^11,12,18^. Both of these putative ligand-release gateways are eligible for the release of the ligand in terms of their free energy and, thus, are physiologically relevant^18^.

The same mechanisms have been proposed based on the structures of several OBPs with a long C-terminus, e.g., ApolPBP from *Antheraea Polyphemus*^10^ and AtraPBP1 from *Amyelois transitella*^21^. However, for some OBPs with a shorter C-terminus, such as AmelPBP1 from *Apis mellifera*^22^, AgamOBP1 from *Anopheles gambiae*, AaegOBP1 from *Anopheles aegypti*^2^, and CquiOBP1 from *Culex quinquefasciatus*^23^, the C-terminal loop folds into the binding cavity to adversely impact ligand binding, without undergoing the random coil-to-helix transition^24^.

Currently, despite much progress, many studies of the OBPs odorant-binding mechanisms have limitations. First, most previous studies focused on the structure and function of the C-terminus or some key residues of OBPs^16^, and they rarely made a concentrated effort to study N-terminal or whole-protein structural changes upon odorant binding. Second, most studies focused on the ejection mechanism of the OBPs, while the processes of odorant binding and OBP activation remain unclear. Third, the basis of the complex sensory system that discriminates thousands of volatile substances at low concentrations^25^ remains a big challenge.

**Figure 2:**
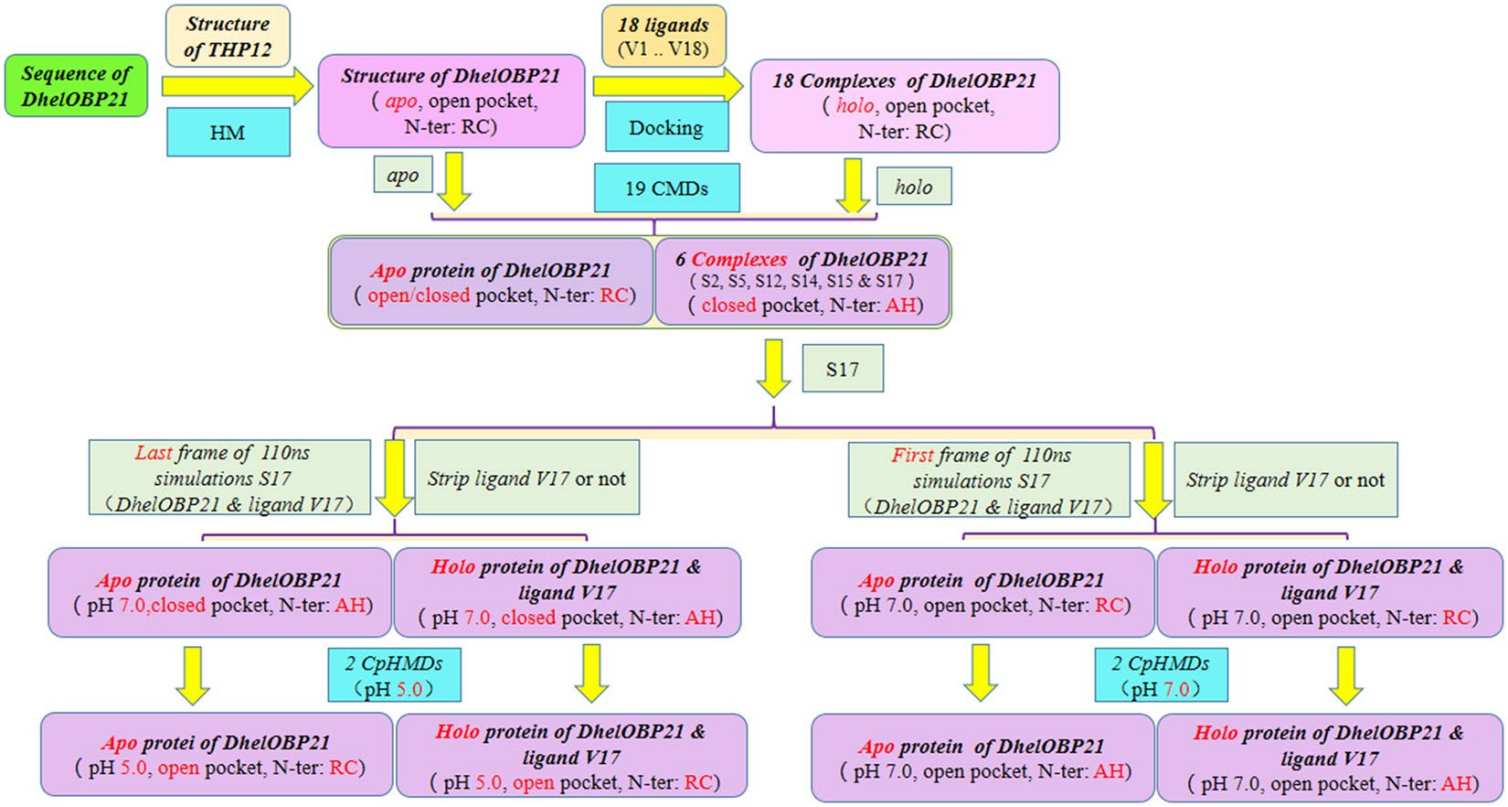
The workflow of this study. HM refers to Homology modelling. RC and AH refer to Random Coil and Alpha Helix, respectively. N-ter refers to N-terminus. CMD and CpHMD refer to Conventional Molecular Dynamics and Constant pH Molecular Dynamics, respectively. S17 refers to the CMD Simulation of DhelOBP21 and (+)-Sativene (Table 1).

To address these issues and gain more insight into the odorant binding mechanism, here we conducted all-atom conventional molecular dynamics (CMD) simulations for the binding of 18 ligands to DhelOBP21, an OBP characterized by our group from *Dastarcus helophoroides*, which is the most important natural enemy of the forest pest *Monochamus alternatus*^26^. The binding free energies were calculated and decomposed to reveal the contributions of each residue in the binding pocket. The results showed that the N-terminus underwent a random coil-to-helix transition during binding. We hypothesized that both pH and odorant binding might contribute to this secondary structural transition. Based on this hypothesis, we conducted four constant-pH molecular dynamics (CpHMD) simulations with different initial states, i.e., an open *apo* pocket and a closed *holo* pocket at pH = 7.0 and pH = 5.0, to explore the course of the structural transition (Fig. 2).

## Results

### The Root-mean-square Deviations (RMSDs) and Root-mean-square Fluctuations (RMSFs)

**Figure 3:**
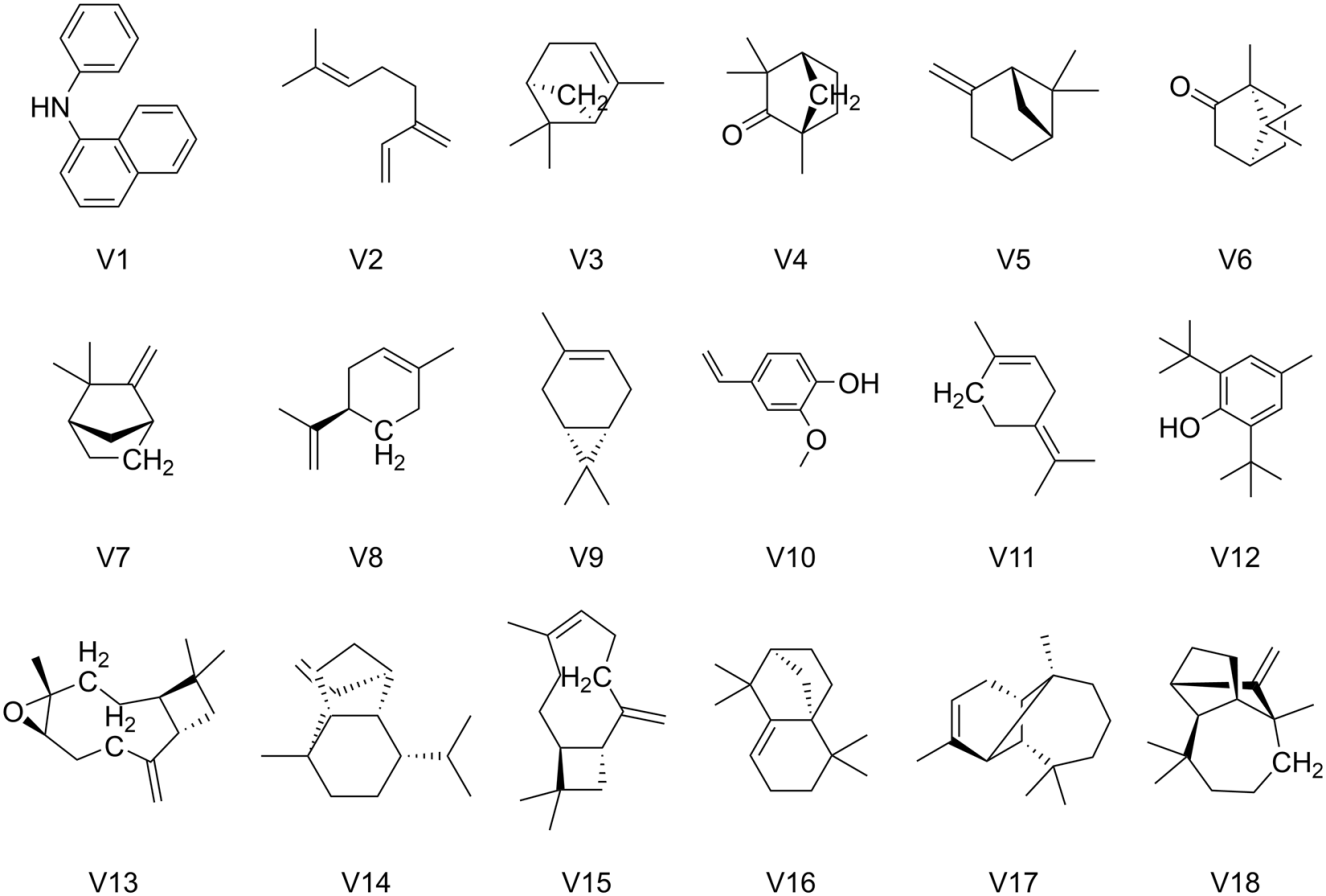
The structures of the 1-NPN (V1) and 17 volatiles (V2 to V18). Refer to Table 1 for details.

According to the RMSD plots of protein main chain in all the 19 systems (*apo* DhelOBP21 and DhelOBP21 bound to 1-NPN and 17 volatiles listed in Fig. 3, respectively) (Fig. S1), it was apparent that DhelOBP21 underwent remarkable conformational shifts ranging from 3 and 6 Å at the beginning of the simulation (closed system with constant-temperature, constant-pressure, NPT ensemble), mostly in the first 40 ns, eliminating the spatial conflicts. All the 19 systems converged in the 110-ns simulations.

Meanwhile, the average RMSF per residue of DhelOBP21 (main chain C_α_, N, and C atoms) showed that sharper fluctuations were found in the N-terminus (4–11 Å), C-terminus (2~5 Å), and α_4_-α_5_ bend region (residues 67 to 77, 2~5 Å), which were observed in all the 19 systems (Fig. S2). In addition to these features, a common peak (1~3 Å) was located in helix α_2_ and its vicinity (residues 15 to 25).

### Secondary structural transition of DhelOBP21

**Figure 4:**
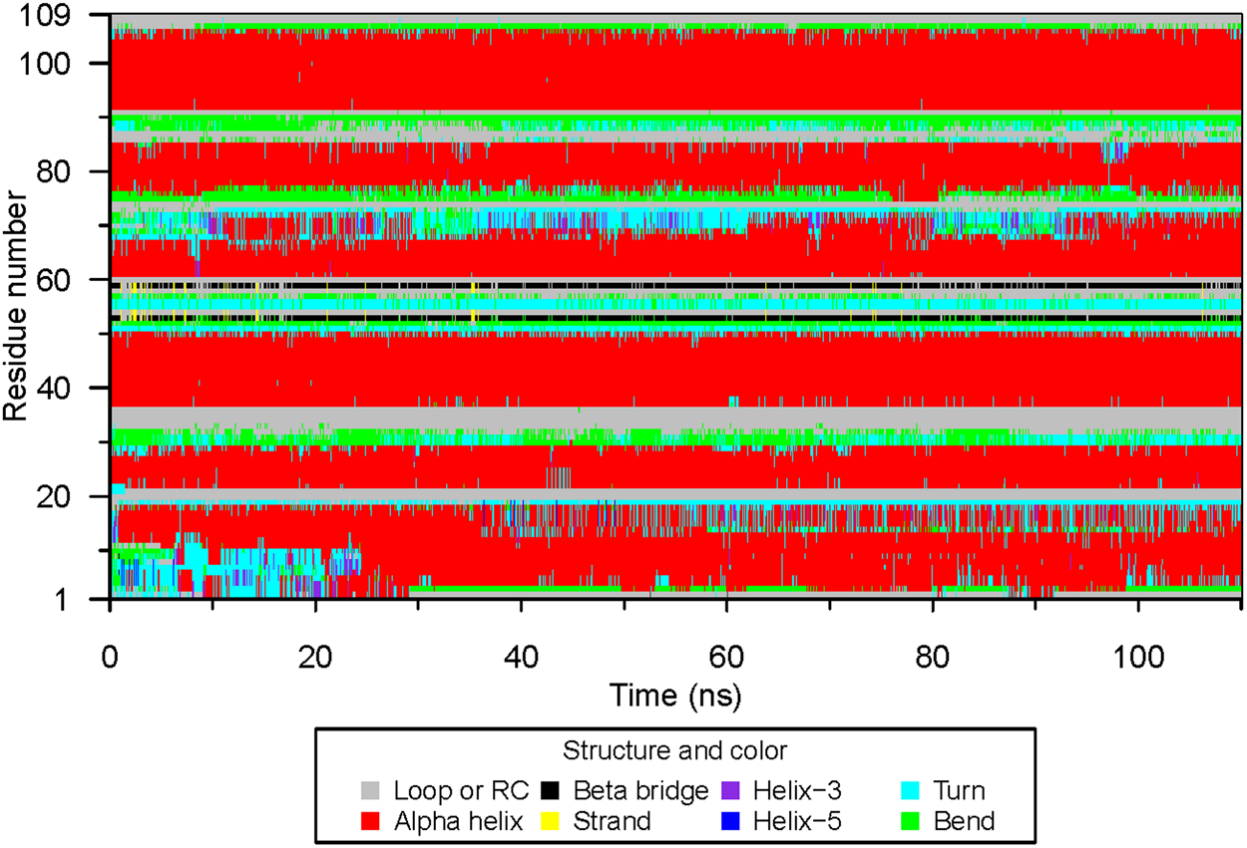
The secondary structural variation of DhelOBP21 in S17 in CMD. Secondary structures were defined by program DSSP, where RC refers to Random Coil. Apparent conformation change started from 7 ns, during which the residues 1 to 10 formed a new Alpha Helix from the Random Coil.

**Figure 5:**
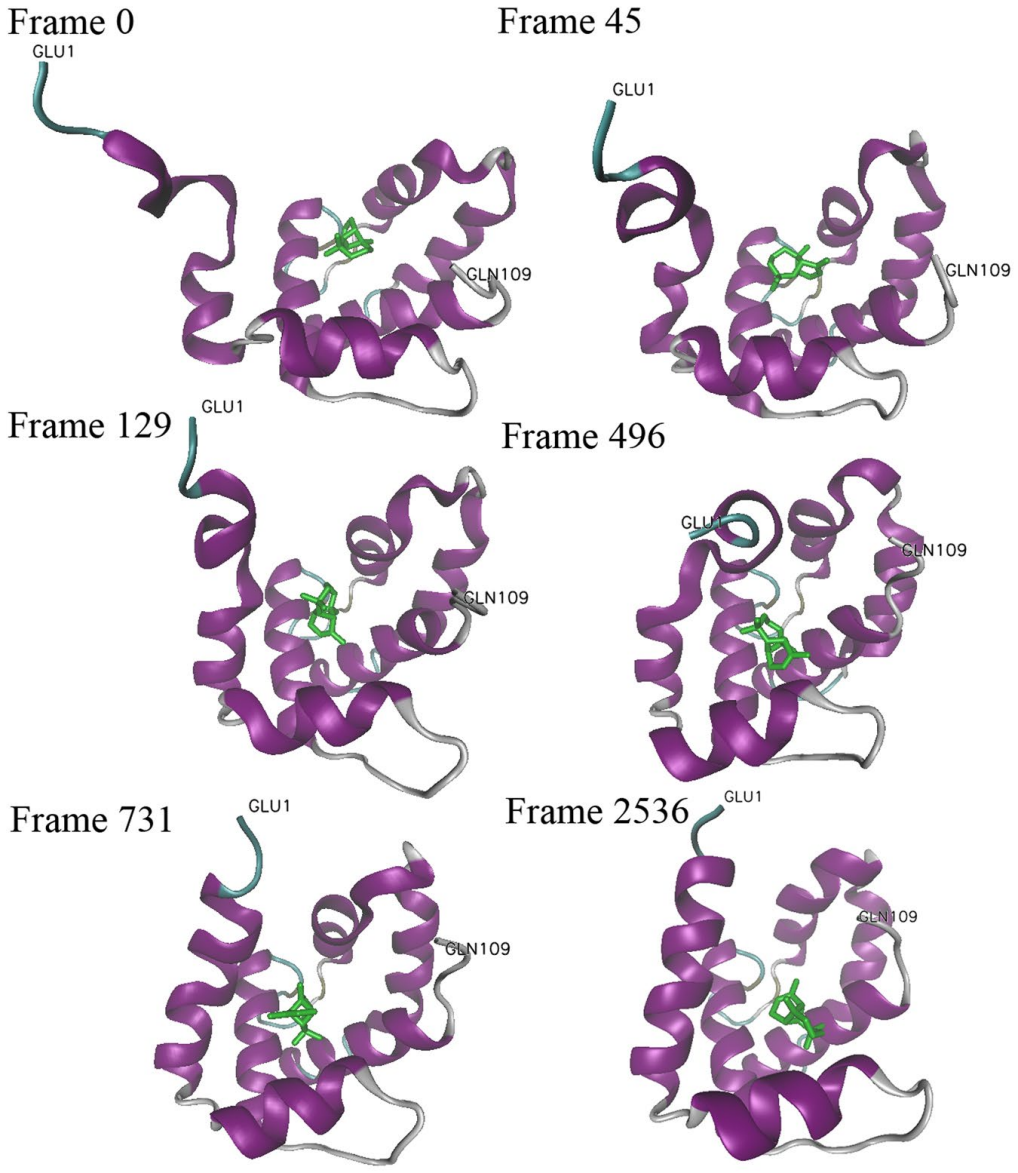
The 6 representative conformations of DhelOBP21 in S17. The (+)-Sativene was colored green. The GLU1 and GLU109 represented the N- and C- termini, respectively. The total frame number was 11000.

We discovered a secondary structural evolution of DhelOBP21 in six of the 19 systems, S2, S5, S12, S14, S15, and S17 (Fig. S3). The proteins in the six complexes experienced a structural transition that the N-terminal random coil transformed gradually into an α-helix (Movies S1–S3). The structural evolution and six representative configurations of S17 are illustrated in Figs 4 and 5, respectively, as this transition occurred firstly in S17.

The simulation started from the initial structure whose N-terminus was elongated as a highly unstructured random coil and the odorant-binding pocket was completely open (Fig. 5, Frame 0). Soon, the N-terminus moved toward the pocket and exhibited a tendency to form an α-helix after 210 ps (Frame 21). Then, the N-terminus became increasingly compact, and it moved rapidly toward the pocket (Frames 45, 129, and 496). After approximately 7.3 ns, a relatively standard α-helix formed completely and sloped toward the cavity (Frame 731). Henceforth, the newly formed helix bent inward and wavered steadily, indicating that it was unstable. Subsequently, the helix bent inward due to the strong attraction of (+)-Sativene (volatile 17, V17), adopting a much more compact conformation (Frames 2536 to 11,000). During this period, apart from the two termini, maximal instability lay in the hinges and loops between the α-helices of DhelOBP21, especially the α_4_-α_5_ loop, which kept swinging and flipping over. Simultaneously, (+)-Sativene rotated inside the cavity, led to non-bonding interactions with helices α_1_, α_2_, α_3_, α_4_, and α_6_.

The aforementioned structural transition occurred in most of the 18 systems. But some of them were unstable, e.g., in S3, residues 3 to 5 of the N-terminus turned into a helix-like conformation that appeared and disappeared transiently and repeatedly. Unfortunately, it failed to form a complete helix and extend to the short α_1_ helix (residues 12 to 17). In addition to this random coil-to-helix transition, conversions among a helix, turn, and bend occurred frequently in the N-terminus in most of the simulations.

### Hydrogen bonding variations in the CMD simulations

An α-helix consists of at least two continuous turns, in which every backbone NH group forms a hydrogen bond to the backbone C=O group of the amino acid located three or four residues earlier along the protein sequence. It is of great importance to analyze the formation of hydrogen bonds to understand the conformational transitions from random coil to helix.

The formation of the new α-helix was highly correlated to these main-chain hydrogen bonds, i.e., bonds between Gln3 and Ile7, Lys4 and Lys8, Glu5 and Ala9, Lys6 and Tyr10, Ile7 and HIE11 (HIS with a hydrogen bond to ε-nitrogen), Lys8 and Lys12, Ala9 and Asp13, Tyr10 and CYX14 (CYS formed a disulfide bond) (Fig. S4). These eight hydrogen bonds led to a random coil-to-helix transition of the N-terminus of DhelOBP21, which resulted in the formation of a four-turn helix.

**Figure 6:**
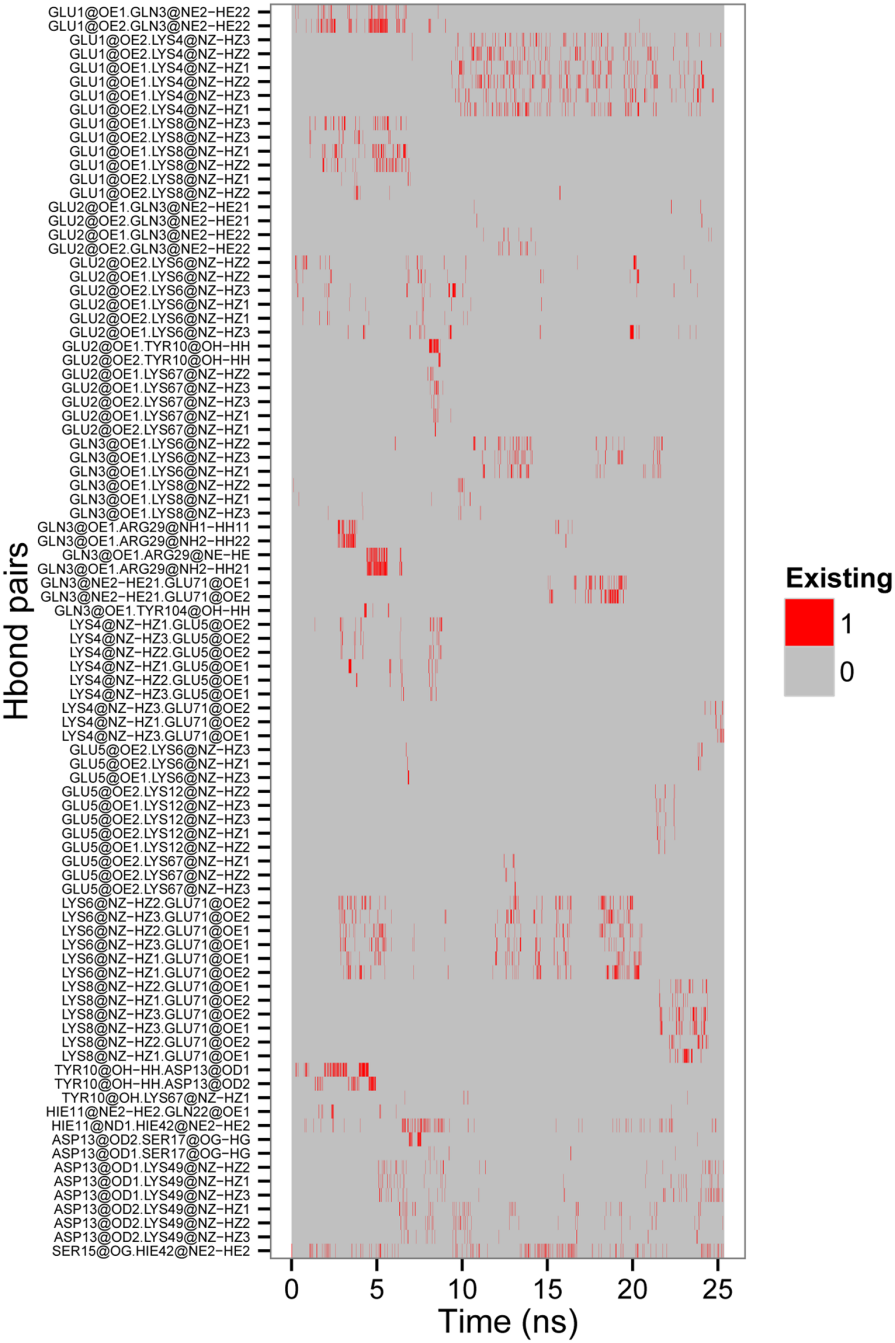
The hydrogen-bonds formed with the side chains of DhelOBP21 in the first 25.36 ns in S17, where 1 refers to the existence of h-bond, and vice versa.

Although the secondary structural transition of DhelOBP21 was directly correlated with the formation and breakage of its main-chain hydrogen bonds, it is fundamentally determined by the variation of the protein side-chain hydrogen bonds. Hence, these H-bond variations in the first 18 N-terminal amino acid residues of DhelOBP21durning complete the random coil-to-helix transition (first 25.36 ns, 2536 frames) were investigated (Fig. 6). There were 20 amino acid residues (Glu1, Glu2, Gln3, Lys4, Glu5, Lys6, Lys8, Tyr10, HIE11, Lys12, Asp13, Ser15, Ser17, Glu22, HIE42, Lys49, Lys67, Arg29, Glu71, and Tyr104) and 91 hydrogen bond pairs left after removing hydrogen bond pairs that occurred fewer than five times among the 2536 frames.

Initially, the distance between the phenolic hydroxyl H atom of Tyr10 and the carboxyl O atom of Asp13 was very short, with a hydrogen bond between them. Then, the side chain of Tyr10 moved toward Asp13 (Frame 23 in S17), leading to a conformational alteration of the main chain. Consequently, the helix-3, initially consisted of Lys12, Asp13, and Cys14, extended to the N-terminus. Meanwhile, the N-terminus (amino acid residues 1–8) tends to form a helix in this region, as three hydrogen bonds formed between carboxyl and amidogen of Glu1 & Lys8, Glu2 & Lys6, and Gln3 & Lys8, respectively.

Later, as the N-terminus rotated, a hydrogen bond formed between Glu1 and Lys8, indicating that the N-terminus was bending toward the pocket. Then, Gln3 moved toward Tyr104 (α_6_), and the carboxyl oxygen of Gln3 formed a transient hydrogen bond with the phenolic hydroxyl group of Tyr10 (Frame 423). Subsequently, hydrogen bonds formed between the O atom (at side chain) of Gln3 and the NH group of Arg29 (α_6_). Furthermore, there were strong hydrogen bonds between the phenolic oxygen of Tyr10 and the two carboxyl oxygens of Asp13 (α_1_), the phenolic oxygens of Tyr10 and Lys67 (α_4_), as well as an intermittent hydrogen bond between the N_ε_H group of HIE11 and the carboxyl oxygen of Glu22. The N_δ_ of His11 formed a hydrogen bond with the N_ε_H group of HIE42 (α_3_), and the carboxyl oxygen of Asp13 hydrogen bonded with the NH group of Lys49 (α_3_). These hydrogen bonds drew the N-terminus toward these helices (α_2_, α_3_, α_4_ and α_6_), i.e., the N-terminus approached the pocket (Frame 538).

Then, many of the formed hydrogen bond at N-terminus were broken, including the six hydrogen bonds between the carboxyl oxygen of Glu1 and the NH group of Lys8 (Frame 675), the four hydrogen bonds between the side chain O atom of Gln3 and the NH group of RG29 (α_2_), the six hydrogen bonds between the NH group of Lys6 and the two carboxyl oxygen atoms of Glu71 (α_4_), the hydrogen bond between the carboxyl oxygen of Gln3 and the phenolic hydroxyl hydrogen atom of Tyr104 (α_6_). Hence, the N-terminus moved away from the pocket, and the first eight amino acid residues gradually formed a helix again. Subsequently, Lys6 moved close to Glu71 (α_1_), and the NH_2_ group of Lys6 formed four hydrogen bonds with the carboxyl oxygen atoms of Glu71 (Frame 715), although these hydrogen bonds broke instantaneously (Frame 720). Then, the sidechain NH_2_ group of Lys6 formed a hydrogen bond with the backbone O atom of Lys67 (α_4_) (Frame 732). At this time, the relatively regular α-helix was formed.

Following the formation of the relatively regular α-helix, Lys6 formed hydrogen bonds with Glu2 and Lys67 (Frame 933), connecting both the N-terminus and helix α_4_. Finally, Lys4 formed a hydrogen bond with Glu71, which not only connected the α_4_ helix and the newly formed α_1_ helix, but also stabilized the α_1_ helix (Frame 2536). Therefore, the hydrogen bonds among these amino acid residues played an important role during the random coil-to-helix transition. Additionally, the hydrogen bonds between the N-terminal (α_1_) and the other four helices (α_2_, α_3_, α_4_, and α_6_) drove the N-terminus toward the pocket and gradually stabilized this conformation.

### The RMSDs and RMSFs of the CpHMD Simulations

The RMSDs and RMSFs of the four CpHMD simulations are shown in Figs S5 and S6, respectively. The RMSDs of the open pocket *holo* (red) and *apo* (blue) proteins at pH 7.0 in the CpHMD simulations (Fig. S5, left) were significantly higher than those of the *apo* (black) protein in the CMD simulations, with differences of 4 Å and 2 Å, respectively. This illustrated that the CpHMD simulations with an explicit solvent model in Amber14 had a much greater impact on the three-dimensional structure of DhelOBP21 than the CMD simulations. This may be due to the continuous sampling and alteration of the protonation states of the titratable residues, which resulted in an unstable protein structure, leading to much higher RMSDs, while there were also much larger fluctuations in the closed pocket *holo* (purple) and *apo* (green) proteins at pH 5.0 (Fig. S5, right).

Additionally, there were severe vibrations in the protein main chain during the first 100 ns relative to their average position. Like the case in the CMD simulations, the largest fluctuations resided in the N- and C-termini, as the fluctuation of the *holo* (purple) protein N-terminus was as high as 12 Å at pH 5.0, which is in accordance with the secondary structural transition of the N-terminus and its movement away from the binding pocket. Apart from the two termini, the fluctuation of the α_4_ helix was also large (>4 Å) at both pH 7.0 and 5.0 (Fig. S6).

### Secondary Structural Transition of DhelOBP21 in the CpHMD Simulations

First, in the simulations of the open pocket *holo* and *apo* proteins, although there was no formation of a relatively complete and regular α-helix in the N-terminus of DhelOBP21, a premature helix formed in the N-terminus of both simulations (Fig. S7A,B).

By contrast, in the simulations of the closed pocket *holo* and *apo* proteins (Fig. S7C,D), partial unwinding occurred in the N-terminus, which were complete and regular α-helix initially. More specifically, in the simulation of the closed pocket *holo* protein, the N-terminus was a regular α-helix and it began to bend inward (at 5 ns, Frame 500), while the first 15 amino acid residues in the N-terminus became almost totally unwound (at 9.8 ns, Frame 980). However, the N-terminus transformed into a premature helix again (at 20 ns, Frame 2000), then unwound (at 50.7 ns, Frame 5070), and the helix formed again from 78 ns (Frame 7800) and remained until 100 ns (Frame 10,000) before finally moving away from the pocket.

### Hydrogen bond variations in the CpHMDs

The variation of a protein’s protonation states is highly correlated to the variation of the hydrogen bonds of the protein, and the changes of the main-chain hydrogen bonds led directly to the secondary structural transition of DhelOBP21 (Fig. S8). In the following sections, we focused on the variation of the side-chain hydrogen bonds of DhelOBP21, and we used the CpHMD course of the *holo* protein at pH 5.0 to illustrate the phenomena (Fig. S9). In this case, there were 88 hydrogen bonds among 26 amino acid residues, i.e., Glu1, Glu2, Gln3, Lys4, Glu5, Lys6, Lys8, Tyr10, HIP11 (HIS with one positive charge while both δ and ε nitrogen atoms protonated), Lys12, Asp13, Ser15, Ser17, Ser18, Gln22, Arg29, Asp36, Glu41, HIP42, Lys49, Asp74, Tyr103, Tyr104, Glu105, Thr107, and Gln109. It is worth noting that those hydrogen bonds occurrence less than 20 times among the 10,000 frames were excluded in the following analysis.

Combined with a visual inspection of the trajectory via VMD (Visual molecular dynamics) and the information in Fig. S8, the variation of the hydrogen-bonding network was as follows. The hydrogen bonds between Asp13 and Lys49 (α_3_), and Tyr10 and Lys67 (α_4_) broke in the beginning. During the first 5 ns, the hydrogen bonds between Glu1 and Tyr103 (α_6_), Lys4 (α_1_, the N-terminus), and Thr107 (α_6_) broke, which led the N-terminus to move away from helices α_3_, α_4_, and α_6_ (Frame 500). Furthermore, both the backbone and side-chain hydrogen bonds between Glu2 and Lys6 were completely broken. This breakage also led to the N-terminus (amino acid residues 1–8) unwinding, turning into a random coil, and moving away from the pocket. Although Lys8 subsequently formed hydrogen bonds with Thr107, these hydrogen bonds broke within the first 20 ns, i.e., the N-terminus moved much farther away from the pocket and the C-terminus. Subsequently, although many hydrogen bonds formed, such as the hydrogen bonds between Glu5 and Asp13, Glu2 and Lys6, Glu1 and Gln3, and Glu1 and Lys4, the N-terminus kept unwinding and moved away from the pocket.

### OBP-volatile Interactions

To comprehend the characteristics of the interactions between DhelOBP21 and the 18 ligands (1-NPN and 17 volatiles), the free binding energies (*G*_GBSA_, generalized Born surface area model and (PB) and *G*_PBSA_, Poisson–Boltzmann surface area model) were calculated for the 18 systems. The Pearson product-moment correlation coefficient (*r*) and Spearman’s rank correlation coefficient (*ρ*) were calculated for *G*_GBSA_ and *G*_PBSA_ (Table S1). The *r* and *ρ* between *G*_GBSA_ and *G*_PBSA_ were 0.840 and 0.841, respectively, which illustrated the high correlation between the MM-PBSA and MM-GBSA.

**Figure 7:**
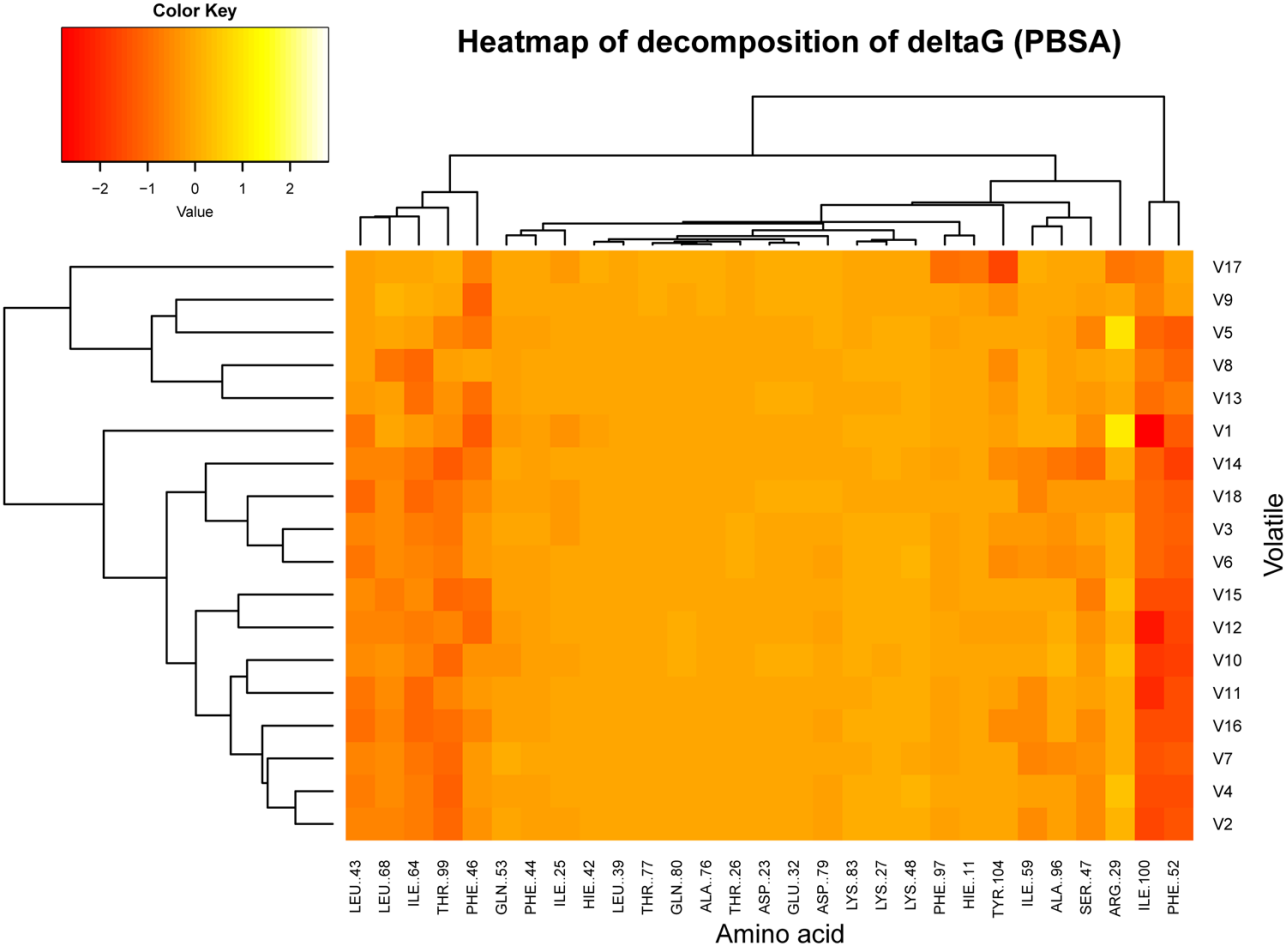
The cluster heatmap of the contributions to delta G (PBSA) per residue in the 18 simulations. In this rectangular array, the rows represent the 18 ligands, and the columns represent the 29 amino acids in the protein pocket within 4.5 Å of the ligands. Each tile was shaded on a color scale to represent the decomposition of delta G (PBSA) of the corresponding residue and ligand, ranging from red to white. Lower delta G value (red) represents a favored ligand-residue interaction and higher value (white) represents an unfavored interaction. Besides, both the volatiles and the residues were clustered with hierarchical cluster analysis based on decomposition of delta G (PBSA) per residue.

**Figure 8:**
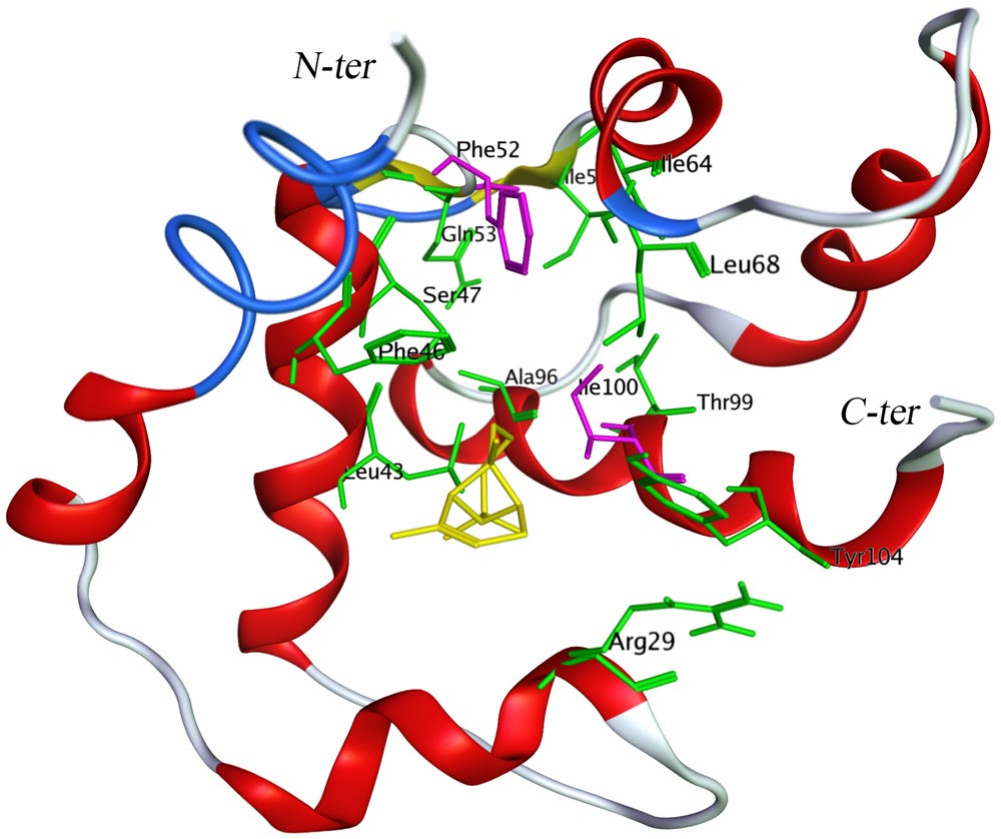
The side view to the DhelOBP21 pocket of S17, the (+)-Sativene was rendered in yellow and the ‘fundamental residues’ Phe52 and Ile100 were colored red, and the ‘selective’ residues (Arg29, Leu43, Phe46, Ser47, Gln53, Ile59, Ile64, Leu68, Ala96, Thr99, and Thr104) were colored green.

Subsequently, the free binding energy was decomposed in two ways. First, it was decomposed per residue to calculate each residue’s contribution to ligand binding. As shown in the cluster heat-map (Fig. 7), the 29 residues in the protein pocket can be hierarchically assigned into three clusters using the Hierarchical Clustering method in R. The minimum cluster on the right consisted of two residues, Phe52 and Ile100, which exhibited high interaction intensities with almost all 18 volatiles, indicating that they were essential for DhelOBP21 to bind volatiles. The left cluster comprised five residues (Leu43, Phe46, Ile64, Leu68, and Thr99), which exhibited varying binding energies with the volatiles, like the other seven residues (Arg29, Ser47, Gln53, Ile59, Ala96, Phe97, and Tyr104) that were not part of this cluster, illustrating the exact discrimination of diverse volatiles was determined mainly by these twelve ‘selective’ residues. Finally, the largest cluster contained the remaining fifteen residues, which generally had much weaker binding affinities for the volatiles (Figs 7 and 8).

Second, the free binding energy was decomposed into different energy items according to the MM-PBSA/GBSA method (Fig. S10). Compared to the solvation free energy components (Delta_G_Solv), which always made a negative contribution to the total free binding energy, gas phase energy contributions (Delta_G_Gas) were always strongly favored to the total free energy in all 18 systems in both the generalized Born (GB) and Poisson–Boltzmann (PB) models. In particular, due to the lack of polar atoms, such as N and O, in volatiles, the electrostatic energy (E_El) was so weak that it could be ignored. Additionally, the polar solvation energy (E_GB and E_PB) also made few contributions to the total free energy in both the GB and PB models. Moreover, in the GB model, the non-polar solvation energy (E_SA_of_GB) was negative and fairly strong, while in the PB model, the dispersion solvation free energy (E_Disper_of_PB) belonging to the non-polar solvation energy was the strongest factor that jeopardized the binding of volatiles. The other component of the non-polar solvation energy (E_NPolar_of_PB) countered the dispersion solvation free energy. Finally, the van der Waals interaction energy (E_vdW) made the largest contribution to the total free energy among these decomposition items.

**Figure 9:**
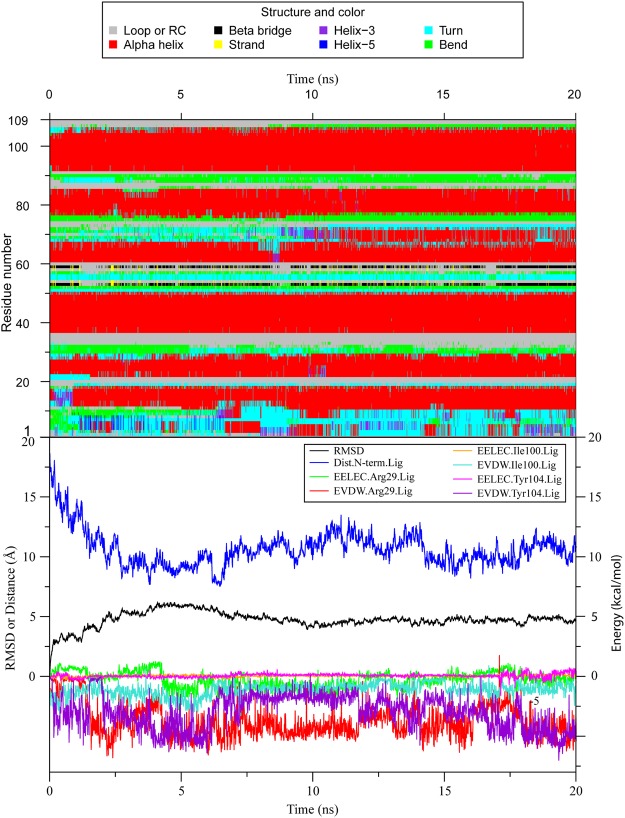
The time evolving secondary structure of the DhelOBP21 in S17 for the first 20 ns (top), and the eight properties of S17 in the first 20 ns (bottom). (1) The time evolving protein back bone Cα RMSD (“RMSD”, black), (2) the geometric center distances between the ligand (+)-Sativene and the N-terminus, i.e. the residues 1 to 15, (“Dist.N-term.lig”, blue), (3~9) the electrostatic and van der Waals interactions between (+)-Sativene and the residues Arg29, Ile100 and Tyr104 (“EELEC.Arg29.Lig”, green, “EVDW.Arg29.Lig”, red, “EELEC.Ile100.Lig”, orange, “EVDW.Ile100.Lig”, cyan, “EELEC.Tyr104.Lig”, violet, “EVDW.Tyr104.Lig”, indigo, respectively).

Third, we focused on the correlations among the secondary structural transition, the energy variation, and the protein-ligand distance. As the remarkable conformation change occurred within the first 20 ns in S17, the non-bounding interactions during this period between the three neighboring residues that interacted most strongly with (+)-Sativene are shown in Fig. 9. From the beginning, the N-terminus gradually moved towards the ligand from its original location approximately 17.5 Å away. At this stage, the electrostatic interactions between the (+)-Sativene and the three residues were nearly zero, while the van der Waals interactions between (+)-Sativene and Arg29 and Tyr 104 increased sharply in the first 7.5 ns. This was accompanied by a decrease of the distance between the geometric centers of the N-terminus and the ligand from 17.5 to 7.5 Å. Simultaneously, the N-terminus became more compact and drew close to the ligand (Fig. 5). After the first 7.5 ns, the helix formed and acted as a lid that covering the binding pocket (Fig. 5, frame 731). Due to the strong interaction between the ligand and N-terminus, as well as Arg29, the ligand moved slightly outward as the van der Waals interaction between the ligand and Tyr104 has severely weakened. After a period of instability during which the tip of the N-terminus bent outward and inward, a regular helix at approximately 25 ns finally formed.

## Discussion

In our earlier study^26,27^, we detected the coil-to-helix transition of the DhelOBP21s with CD analysis. The purpose of this research was to find the sequence which experiences such transition and illuminate the detailed molecular mechanism. After observing a random coil-to-helix transition with MD simulation, we focused on the characteristics of DhelOBP21, the volatiles, and the structural transition of DhelOBP21 to determine the underlying recognition mechanism between DhelOBP21 and the volatiles.

### The Selectivity and Promiscuity of the Binding between Dhelobp21 and the Volatiles

Odorant molecules share many characteristics, such as a low molecular weight, moderate or high volatility, and a relatively high hydrophobicity. Some compounds smell the same but have different structures, e.g., hydrogen sulfide and decaborane, while others smell different but have the similar structures, e.g., ferrocene smells “spicy” while nickelocene has an “oily-chemical” smell^28^, and their high structural diversity leads to the complexity of such odors. A variety of OBPs bind and recognize many odorants, as one odorant can bind to several OBPs, which is defined as promiscuity^29^. However, some OBPs can only interact with one or a few odorants. Our study found that the selectivity of OBPs might correlate to the varieties of the free binding energy contributions of 29 key amino acid residues. Both Phe52 and Ile100 showed a strong binding affinity for almost all the 18 volatiles, indicating that these two residues played a fundamental role in the OBP-volatile binding and recognition process, and they may determine the promiscuity of DhelOBP21. Twelve other residues, i.e., Arg29, Leu43, Phe46, Ser47, Gln53, Ile59, Ile64, Leu68, Ala96, Phe97, Thr99, and Tyr104, exhibited a diverse affinity for the 18 volatiles and, hence, were called selective amino acid residues. They may be associated with the selectivity and specificity of OBPs for thousands of volatiles.

### Characteristics of these Volatiles

**Figure 10:**
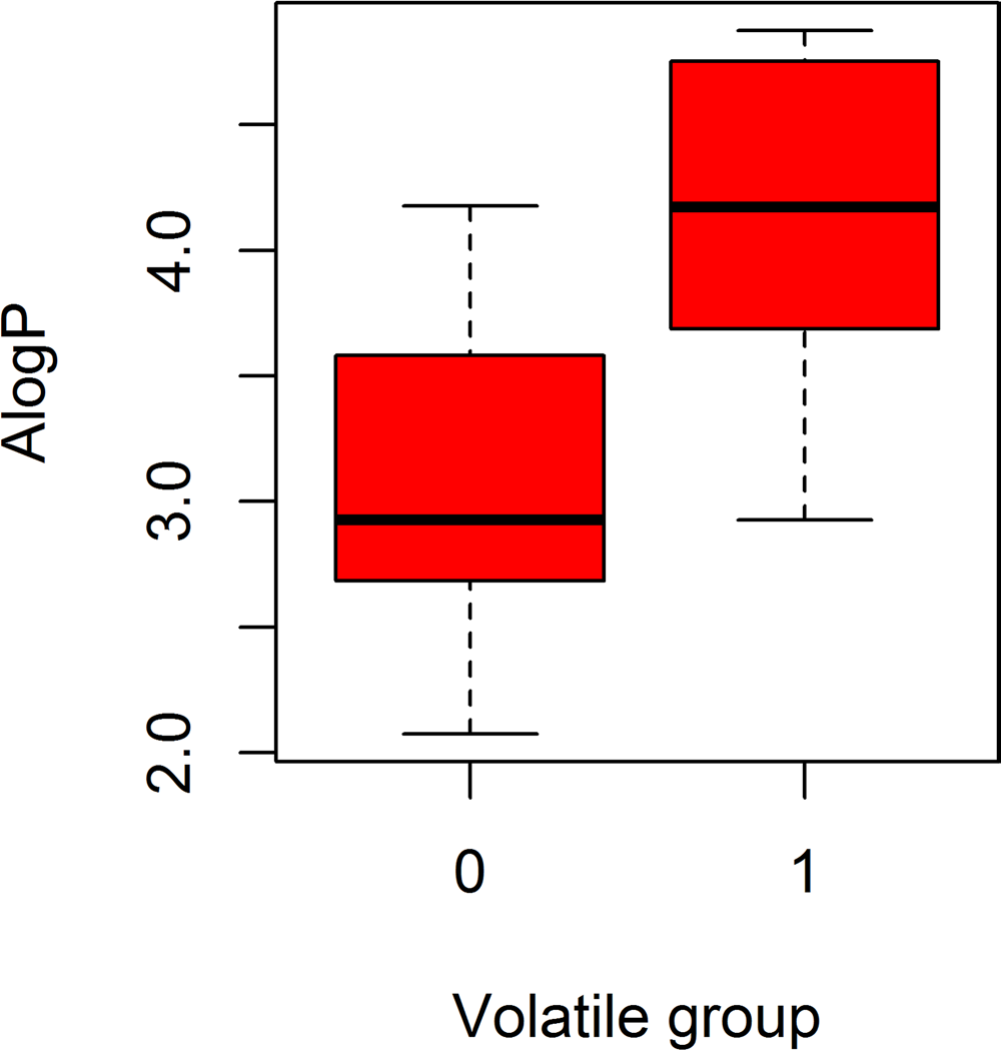
Bar plot of the two volatile groups’ ALogP. Volatile group 1 consisted of ligands (V2, V5, V12, V14, V15, and V17) which a coil-to-helix transition occurred in its corresponding simulation. And group 0 consisted of the remaining 11 volatiles without ligand V1.

To investigate whether there was a significant physico–chemical property difference between the six volatiles (Group 1, S-(−)-Limomeme, 3-Canene, 2-Methoxy-4-vinylphenol.Kosher, (−)-Caryophyllene oxide, (−)-Fenchone, (+)-Sativene, corresponding to V2, V5, V12, V14, V15, & V17, respectively, Table 1) and the other 11 ligands (Group 0), a Wilcoxon test (*U*-test) was applied to 52 properties (Table S2). The only difference was that the AlogP (LogD_7.4_), i.e., the mean of the AlogP of Group 1 (4.1) was significantly higher than that of Group 0 (3.1) (Fig. 10). However, since the *p*-value was 0.016, we speculated that not only these six volatiles, but also some of the others, could induce a random coil-to-helix transition with a longer simulation, and that the more hydrophobic the volatile, the greater the possibility of inducing the transition.

**Table 1:** Main properties of the fluorescent probe 1-npn and 17 volatiles.

ID	PubChem ID	Ligand Name	Molecular Weight	Molecular Volume	Molecular Fractional Polar SASA	LogD	pH 7.4 WT	Corresponding Simulation
V1	7013	1-NPN	219.28	150.23	0.07	4.288	NA	S1
V2	31253	S-(−)-Limomeme	136.23	113.53	0	3.687	70.37	S2
V3	82227	Terpinolene	136.23	114.56	0	2.872	53.73	S3
V4	16213045	(+)-α-Pinene	152.23	119.7	0.14	2.497	49.72	S4
V5	10290825	3-Canene	136.23	112.5	0	2.926	49.12	S5
V6	2537	(+)-β-Pinene	152.23	120.04	0.14	2.075	49.11	S6
V7	6616	(−)-Isolongifolene	136.23	112.84	0	2.926	49.02	S7
V8	439250	Myrcene	136.23	112.5	0	3.502	43.8	S8
V9	26049	β-Caryophyllene	136.23	116.96	0	2.872	42.1	S9
V10	332	Butylated hydroxytoluene	150.17	103.92	0.18	2.122	31.18	S10
V11	11463	Camphor	136.23	113.87	0	3.643	30.54	S11
V12	31404	2-Methoxy-4-vinylphenol	220.35	181.78	0.12	4.874	29.55	S12
V13	1742210	(+)-α-Longipinene	220.35	183.84	0.06	3.519	20.8	S13
V14	71448981	(−)-Caryophyllene oxide	204.35	169.78	0	4.222	16.6	S14
V15	5281515	(−)-Fenchone	204.35	168.06	0	4.753	6.91	S15
V16	11127402	Camphene	204.35	168.75	0	4.077	2.72	S16
V17	42608167	(+)-Sativene	204.35	171.84	0	4.123	0.42	S17
V18	16217634	(+)-Longifolene	204.35	168.41	0	4.177	0.14	S18

Note: V1 refers to Volatile 1, and S1 refers to the corresponding simulation of DhelOBP21 and V1. The others are termed in the same fashion. S0 refers to the simulation of apo state DhelOBP21. pH 7.4 WT refers to the 1/K_i_*1000 which was the binding affinity between the DhelOBP21(wild type) and volatile^16^. SA and SASA refer to surface area and solvent available surface area, respectively.

### The Secondary Structural Transition

Strict random coil-to-helix transition occurred in six of the 19 simulations, i.e., S2, S5, S12, S14, S15, S17. The newly formed helix covered the binding pocket and stabilized the volatiles. The N-terminus moved toward the pocket in the other six systems, namely, S4, S7, S9, S10, S16 and S18. Particularly, the N-terminus transformed into helix-like conformations in S10 and S16. Such observations were elaborately studied in the CpHMD simulations, which showed that the N-terminus of the open pocket of the *apo* and *holo* states of DhelOBP21 transformed partially or wholly into an α-helix at pH 7.0. Additionally, the newly formed helix of the closed pocket *apo* and *holo* states of DhelOBP21 unwound partially at pH 5.0. Additionally, this coil-to-helix transition was in accordance with the circular dichroism (CD) assay in which the helix content did varied significantly with ligand binding and pH change^27^.

**Figure 11:**
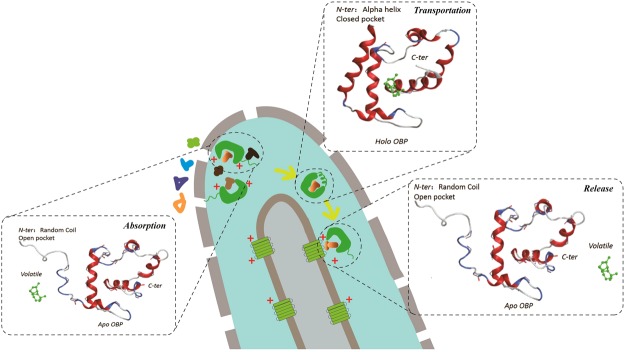
Schematic representation of the hypothesis “Both pH- and volatile binding-dependent coil-to-helix transition of N-terminus of a Minus-C OBP” in an insect olfactory hair.

Based on these investigations, we hypothesized that there were both pH- and volatile binding-dependent coil-to-helix transitions of the N-terminus of a Minus-C OBP (Fig. 11). The *apo* state of an OBP possesses an open pocket in which the N-terminus is partly or fully extended as a random coil at an acidic pH. Once the volatile enters the lymph and is close to the OBP’s cavity, it is captured and roams inside the pocket by adjusting its position via interacting strongly with several pocket residues, especially the essential residues Phe46 and Ile100. Only some of those ligands can interact effectively with both the N-terminus and the selective residues lining the pocket (Leu43, Phe46, and Tyr104) through van der Waals interactions, which lead to the rotation and inward movement of the N-terminus. When the pH increases to neutrality, these titratable residues become deprotonated, and more hydrogen bonds are formed between the hydrogen-bonding groups of the residues because of the strong electrostatic and van der Waals interactions between the N-terminus and volatile. Gradually, the random coil transforms into a helix at neutral pH and covers the pocket as a lid, preventing the volatile’s release. When these volatile-protein complexes are transferred to the odorant receptor, the pH decreases, and the interaction between the OBP and the odorant molecule is altered, which leads to the breakdown of the hydrogen-bonding network, especially the hydrogen bonds at the N-terminus. Hence, the α-helix unwinds, and the pocket opens gradually. Finally, the volatile is released from the OBP and interacts with the odorant receptor, which fires downstream impulses along the olfactory nerves.

The activation of the OBP does not only depend on the binding free energy of the volatile, but also on the intrinsic characteristics of both the volatile and the protein. During the interaction between a volatile and the binding cavity, the pocket becomes more compressed, and several selective residues of the pocket (Fig. 8) play an important role in the discrimination of these volatiles.

Above hypothesis is reasonable, as the regular α_1_ helix of BmorPBP1^B^ (residues 1 to 11) is transformed partly into a random coil at pH 4.9^10,16,20^ (Fig. 1). However, our conclusion differs from the theories mentioned in the introduction section, mainly in two ways. First, as the Minus-C DhelOBP21 possesses a short C-terminus, its N-terminus, not its C-terminus, underwent the coil-to-helix transition. Second, unlike BmorPBP1, whose C-terminus transforms into a helix that is inserted into the binding pocket at an acidic pH, the N-terminus of DhelOBP21 unwinds into a random coil and moves away from the pocket at pH 5.0 forming a helix and moves closer to the pocket and acts as a lid at pH 7.0.

In conclusion, based on previous studies and our investigation, we proposed that there is a both pH- and volatile binding-dependent random coil-to-helix transition of the N-terminus of a Minus-C OBP. The more hydrophobic the ligand, the easiness coil-to-helix transition occurs. Additionally, the selectivity and promiscuity of an odorant may be determined by both the volatiles and the OBP, especially the selective residues that we identified in the binding pocket, rather than the binding affinity of the volatiles. The odorant recognition and discrimination of OBPs that we have identified therefore assists in our understanding of the basis of complex sensory system.

## Methods and Materials

### CMD simulations of DhelOBP21 and the 18 ligands

The three-dimensional structure of DhelOBP21 was constructed with homology modeling. THP12 (Hemolymph Protein from Mealworm Beetle *Tenebrio Molitor*) was used as template (PDB: 1C3Z)^30^. The binding modes between DhelOBP21 and the 18 ligands (Fig. 3 and Table 1), including the fluorescence probe 1-NPN and 17 volatiles, were obtained through molecular docking, as reported in our previous investigation^26^. Prior to the simulations, the structure of the DhelOBP21 (*apo*) was titrated and optimized with the program ‘Protonate3D’ embedded in Molecular Operating Environment (MOE, version 2012). Partial charges were calculated with Amber ff99SB force field.

These complexes were solvated in an octahedral TIP3P water box with 10 Å between the solute atoms and the boundaries. Counter ions were added to neutralize the total charge of the system. The Mbondi2 model was chosen as the GB model. The force field was set to Amber 03.r1. The time step was 2 fs, and the non-bonded cutoff for electrostatic interactions was 12 Å for particle mesh Ewald^31^ calculations, and ig was set to −1 to avoid synchronization artifacts.

The simulations were performed with pmemd.MPI in the parallel version of Amber 14^32^, which comprised four stages: energy minimization, heating (NVT, number of particles (N), system volume (V) and temperature (T) taken as constant), equilibration (NPT, number of particles (N), system pressure (P) and temperature (T) taken as constant), and production MD (NPT). In the energy minimization procedure, the protein complexes were first relaxed with 500 steps of steepest descent and 500 steps of conjugate gradient minimization. Furthermore, the positions of the protein’s heavy atoms were restricted with a force constant of 50 kcal/mol·Å^2^. SHAKE^33^ was shut down to optimize the carbon-hydrogen bond. The second minimization (500 steps of steepest descent and conjugate gradient, respectively) was performed with the force constant reduced to 10 kcal/mol·Å^2^. The third minimization (4500 steps of steepest descent and 500 steps of conjugate gradient) was conducted without any restraint to achieve an optimal structure. In all the subsequent simulations, the SHAKE algorithm was enabled to speed up the computation. Then, with the Langevin thermostat, these systems were heated gradually from 0 K to 300 K within 50 ps, with a restraint on the protein’s heavy atoms. To equilibrate the system further, pressure coupling was adopted to maintain a constant pressure of 1 bar in the next 50-ps simulation at 300 K. In addition, a slight position restraint, in which the force constant was 2 kcal/mol·Å^2^, was applied to the heavy atoms of the protein both in the heating and equilibration procedures to prevent it from undergoing a significant conformational change. During the 10-ns production run, constant temperature (300 K) and pressure (1 bar) coupling was conducted without any constraint. Related information such as coordinates, velocity, pressure, and temperature, were dumped into the corresponding trajectories and output files every 5000 steps (10 ps). Subsequently, 100-ns restarting simulations were conducted after the first 10 ns under the same conditions.

For convenience, 1-NPN was termed volatile 1 (V1), and the 17 volatiles were sorted in descending order according to their binding affinities (indicated by 1/*K*_i_*1000)^26^ to wild-type DhelOBP21, and termed V2–V18, respectively. Then, the corresponding single-simulation systems were termed S1–S18 (e.g., S1 refers to the simulation of DhelOBP21 and 1-NPN). In addition, S0 refers to the simulation of the *apo* protein. Please refer to Table 1 for more details.

To precisely define protein structural changes, structures were extracted every 10 frames, which formed a total of 11,000 frames (110 ns). The structures were subjected to the program DSSP^34^ to compute accurate secondary structures. S14 and S17 were rerun for 110 ns from the beginning with the same parameters to validate the repeatability of the structural transition.

### Cphmd Simulations of the Apo/Holo and Open/Closed Pocket of Dhelobp21 With (+)-Sativene

Unlike the conventional molecular simulation, which assigns a fixed protonation state to the titratable residues, the CpHMD is a more advanced method that employs Monte Carlo sampling of the Boltzmann distribution of protonation states during the molecular simulation. Here we employed the CpHMD method with explicit solvent model implemented in Amber14^35^.

### Cphmd Simulations of the Apo/Holo (With (+)-Sativene) and Open/Closed State of Dhelobp21 at pH 7.0/5.0

The structure of the open form DhelOBP21 was extracted from the first frame of the trajectory of S17 (production MD), in which the N-terminus was a random coil and far away from the pocket. In contrast, the closed form DhelOBP21 was the last frame of the trajectory of S17 (production MD), in which the N-terminus was a regular helix that was close to the pocket. The ligand was stripped from the *holo* protein and the remaining protein was called the *apo* protein. Hence, there were four simulations derived from the two combinations, i.e., the *apo* protein, open pocket (at pH = 7.0); the *holo* protein, open pocket (at pH = 7.0); the *apo* protein, closed pocket (at pH = 5.0, with initial conformation from a state of pH = 7.0); and the *holo* protein, closed pocket (at pH = 5.0, with initial conformation came from a state of pH = 7.0) (Fig. 2).

The four simulations were conducted as follows. First, all the hydrogen atoms were removed and assigned subsequently with tleap. Three kinds of titratable residues, Asp, Glu and His (HID or HIE), were renamed to Asp, Glu, and HIP, respectively. Third, the Cys in the S–S bond was renamed to CYX, and the two S–S bonds were explicitly defined in the PDB file. Fourth, the initial protonation states of these 19 titratable residues were recorded in the cpin file. Finally, the non-bonded cutoff for the GB calculations was 30 Å, and the concentration of mobile counterions in solution was 0.1 M.

For better comparison, much effort was made to keep the framework of the CpHMD simulations in line with the CMD simulations. The routine also contained four stages like the CMD simulations, i.e., energy minimization, heating (NVT), equilibration (NPT), and production MD (NPT). The energy minimization, heating, and equilibration phases were the same as those in the CMD simulations, except for two differences. One was that the position constraint was applied to both the heavy atoms of the protein and ligand, rather than only the heavy atoms of the protein in the CMD simulations. The second was that the CpHMD simulations were implemented after the heating stage, and they would attempt to change protonation states every five steps, while the solvent pH was set to 5.0 and 7.0. The simulation time was 100 ns.

### Hydrogen bond calculation

Intramolecular and intermolecular hydrogen bonds were calculated with the program CPPTRAJ in Amber14 with the default standard, i.e., the distance between the hydrogen bond donor and receptor atom should be no greater than 3 Å, and the angles of the donor atom, the polar hydrogen of the donor, and the receptor atom should be greater than 135°. Both the hydrogen bonds in the main chain and side chain were analyzed, and the corresponding times during which the hydrogen bond networks evolved were investigated carefully.

### Binding Free Energy Calculation and Energy Decomposition of MM-PBSA/GBSA

The MM-PBSA/GBSA method, which has been used widely to evaluate the binding free energy of protein-ligand complexes^36,37^, was used to evaluate the binding affinity between DhelOBP21 and each of the 18 volatiles.

In the MM-PBSA/GBSA method, the binding free energy (*G*_bind_) of forming a protein-ligand complex was computed using formulae (1) to (4):

1 $${\rm{\Delta }}{G}_{{\rm{bind}}}={G}_{{\rm{complex}}}-{G}_{\mathrm{receptor}}-{G}_{{\rm{ligand}}}$$

2 $${\rm{\Delta }}{G}_{{\rm{bind}}}={\rm{\Delta }}{G}_{{\rm{gas}}}+{\rm{\Delta }}{G}_{{\rm{sol}}}={\rm{\Delta }}H-T{\rm{\Delta }}S\approx {\rm{\Delta }}{E}_{{\rm{MM}}}-T{\rm{\Delta }}S+{\rm{\Delta }}{G}_{{\rm{sol}}}$$

3 $${\rm{\Delta }}{E}_{{\rm{MM}}}={\rm{\Delta }}{E}_{{\rm{internal}}}+{\rm{\Delta }}{E}_{{\rm{electrostatic}}}+{\rm{\Delta }}{E}_{{\rm{vdw}}}$$

4 $${\rm{\Delta }}{E}_{{\rm{sol}}}={\rm{\Delta }}{E}_{{\rm{PB}}/{\rm{GB}}}+{\rm{\Delta }}{E}_{{\rm{SA}}}$$

Because the complex, protein, and ligand structures are extracted from the same trajectory, the binding free energy (*G*_bind_) was calculated approximately by the energy difference of the complex (*G*_complex_), receptor (*G*_receptor_), and ligand (*G*_ligand_)^38^. In contrast, the binding free energy (Gibbs free energy, *G*_bind_) was calculated by adding the gas phase energy (*G*_gas_) and solvation free energy (*G*_sol_), or it was divided into two portions: enthalpy terms (*H*), which consist of molecular mechanics energy in a vacuum (*E*_MM_) and the solvation free energy (*G*_sol_), and entropy terms with constant temperature (*T*Δ*S*)^39^. Moreover, the molecular mechanics energy (*E*_MM_) was decomposed into bonded energy (*E*_internal_), electrostatic energy (*E*_electrostatic_), and van der Waals interaction energy (*E*_vdw_). Finally, the free energy of solvation (*G*_sol_) was the sum of the polar solvation energy (*E*_PB/GB_) estimated from either the PB^40^ or GB methods^41^, while the non-polar solvation energy (Δ*E*_SA_) was obtained from a fast LCPO algorithm^42^. Here, the entropy contribution was not involved in the calculation, as all these ligands bound to the same protein and a normal mode analysis may not always improve the accuracy of free energy calculation.

After consulting several previous studies^43–50^, 100 snapshots were extracted evenly from the last 50-ns trajectory for the MM-PBSA/GBSA calculation of each complex. We finally employed the GB model (referred as GB^OBC2^) proposed by Onufriev *et al*. (2004) in which α, β, and γ are 1.0, 0.8, and 4.85, respectively, while we set the ionic strength to 0.1 M for the PB calculation and the salt concentration to 0.1 M for the GB calculation. Meanwhile, we decomposed the free binding energy of the MM-PBSA/GBSA to account for the free energy contribution of key residues in the protein pocket^51,52^ (Fig. 7).

## Electronic supplementary material

Movie S1

Movie S2

Movie S3

Supplementary Appendix
