# Supplementary material for: Deciphering the Odorant Binding, Activation, and Discrimination Mechanism of Dhelobp21 from Dastarus Helophoroides
Source: Sci Rep. 2018 Sep 10;8:13506. doi: 10.1038/s41598-018-31959-5 (PMC6131269; doi:10.1038/s41598-018-31959-5)
Supplement: Supplementary file 4 — Supplementary Appendix [file 41598_2018_31959_MOESM4_ESM.pdf]

Supplementary Appendix

Deciphering the Odorant Binding, Activation, and Discrimination Mechanism of Dhelobp21 from *Dastarus Helophoroides*

GUANG-QIANG YU[1,2], DONG-ZHEN LI[1], YU-LIN LU[2], YA-QI WANG[2], DE-XIN KONG[2]*, MAN-QUN WANG[1]*

1. Hubei Insect Resources Utilization and Sustainable Pest Management Key Laboratory, College of Plant Science and Technology, Huazhong Agricultural University, Wuhan 430070, P. R. China

2. Agricultural Bioinformatics Key Laboratory of Hubei Province, College of Informatics, Huazhong Agricultural University, Wuhan 430070, P. R. China

**Corresponding author. Address: College of Plant Science and Technology, Huazhong Agricultural University, Wuhan 430070, P.R.China. Tel.: (0086) 13627126839. E-mail: mqwang@mail.hzau.edu.cn (M.-Q Wang) ; dxkong@mail.hzau.edu.cn (D.-X. Kong)*

Table of Contents

Table S1. Theoretical calculated ΔG (kcal/mol).	p.3
Table S2. All the 52 properties of the 1NPN and 17 volatiles.	p.4
Figure S1. The RMSD of the protein main chain Cα of the 19 simulations.	p.10
Figure S2. Average RMSF per residue of the protein backbone heavy atoms (Cα, N, C) of the 19 CMD simulations.	p.11
Figure S3. The secondary structural evolution of the DhelOBP21 in 19 CMD simulations.	p.12
Figure S4. The hydrogen's evolution in the main chain of DhelOBP21 in S17 during the 110 ns CMD.	p.13
Figure S5. The RMSD of the protein main chain heavy atoms in the 4 CpHMD simulations.	p.14
Figure S6. The RMSF of the protein main chain heavy atoms in the 4 CpHMD simulations.	p.14
Figure S7. The secondary structure evolution of the protein in the 4 CpHMD simulations.	p.15
Figure S8. The main chain hydrogen bond evolution of the holo protein (with V17) with a closed pocket initially at pH 5.0.	p.16
Figure S9. The sidechain hydrogen bond evolution of the holo protein (with V17) with a closed pocket initially at environmental pH 5.0.	p.17
Figure S10. The decomposition of two kinds of binding free energy (MM- PBSA/GBSA).	p.18
Movie S1. The time evolving trajectory of the S17 for the 110ns (1100 frames).	p.19
Movie S2. The time evolving trajectory of the S17 for the first 10ns (1000 frames).	p.19
Movie S3. The time evolving trajectory of the S14 for the 110ns (1100 frames).	p.19

(Note that Movie S1, S2, S3 were provided as individual files and not shown in this file as their sizes were extraordinarily large.)

Table

Table S1. Theoretical calculated ΔG (kcal/mol).

ID	ΔG_{GBSA}	ΔG_{PBSA}	ID	ΔG_{GBSA}	ΔG_{PBSA}
**V1**	-23.3542	-5.4683	**V10**	-21.1043	-4.6116
**V2**	-26.6872	-11.4570	**V11**	-26.7345	-12.1374
**V3**	-24.0340	-9.5857	**V12**	-29.3592	-9.7538
**V4**	-25.6946	-7.2546	**V13**	-26.0427	-9.0872
**V5**	-22.1461	-7.3297	**V14**	-36.8714	-16.5183
**V6**	-26.6899	-7.4012	**V15**	-27.9577	-10.6119
**V7**	-26.5886	-11.5857	**V16**	-31.7960	-11.9070
**V8**	-23.0255	-9.1875	**V17**	-26.3883	-9.5113
**V9**	-21.9465	-6.2332	**V18**	-33.8549	-12.0302

Table S2. All the 52 properties of the 1NPN and 17 volatiles. V1 means Volatile 1, and S1 means the corresponding simulation of DhelOBP21 and V1. The others are termed in the same fasion. “pH7.4 WT” and “pH5.0 WT” refer to the 1/K_i*1000 of the DhelOBP21 against 17 volatiles at pH 7.4 and 5, respectively. These binding affinity data were collected from our previous paper[1]. Note that the residue was numbered from 21 in our previous paper (e.g. S67A) but from 1 in this paper. SA and SASA refer to surface area and solvent available surface area, respectively.

ID	pubchem ID	Ligand Name	Molecular Weight	Molecular Volume	Molecular SurfaceArea	Molecular Polar SASA	Molecular Fractional Polar SASA	Molecular SAVol	ALogP
**V1**	7013	1-NPN	219.28	150.23	206.92	31.74	0.07	376.07	4.288
**V2**	31253	S-(-)-Limomeme	136.23	113.53	172.59	0	0	302.31	3.687
**V3**	82227	Terpinolene	136.23	114.56	151	0	0	258.92	2.872
**V4**	16213045	(+)-α-Pinene	152.23	119.7	165.91	43.49	0.14	261.61	2.497
**V5**	10290825	3-Canene	136.23	112.5	149.18	0	0	257.91	2.926
**V6**	2537	(+)-β-Pinene	152.23	120.04	165.56	43.49	0.14	261.61	2.075
**V7**	6616	(-)-Isolongifolene	136.23	112.84	147.95	0	0	257.91	2.926
**V8**	439250	Myrcene	136.23	112.5	159.03	0	0	286.39	3.502
**V9**	26049	β-Caryophyllene	136.23	116.96	151.08	0	0	258.92	2.872
**V10**	332	Butylated hydroxytoluene	150.17	103.92	159.3	58.18	0.18	291.47	2.123
**V11**	11463	Camphor	136.23	113.87	162.69	0	0	288.38	3.643
**V12**	31404	2-Methoxy-4-vinylphenol.Kosher	220.35	181.78	269.42	52.15	0.12	360.29	4.875
**V13**	1742210	(+)-α-Longipinene	220.35	183.84	233.04	22.68	0.06	337.13	3.519
**V14**	71448981	(-)-Caryophyllene oxide	204.35	169.78	211.22	0	0	324.33	4.222
**V15**	5281515	(-)-Fenchone	204.35	168.06	229.32	0	0	350.70	4.753
**V16**	11127402	Camphene	204.35	168.75	222.73	0	0	321.14	4.077
**V17**	42608167	(+)-Sativene	204.35	171.84	220.78	0	0	323.24	4.123
**V18**	16217634	(+)-Longifolene	204.35	168.41	217.73	0	0	322.23	4.177

ID	Molecular Solubility	Molecular Polar Surface Area	Molecular Fractional Polar Surface Area	Molecular SAVol	LogD	pH7.4 WT	pH5.0 WT	H_Count	C_Count	N_Count	O_Count
**V1**	-5.19	12.03	0.058	376.07	4.288	None	None	13	16	1	0
**V2**	-2.89	0	0	302.31	3.687	70.37	45.37	16	10	0	0
**V3**	-2.43	0	0	258.92	2.872	53.73	29.52	16	10	0	0
**V4**	-2.41	17.07	0.102	261.61	2.497	49.72	36.93	16	10	0	1
**V5**	-2.41	0	0	257.91	2.926	49.12	36.8	16	10	0	0
**V6**	-2.41	17.07	0.103	261.61	2.075	49.11	38.94	16	10	0	1
**V7**	-2.44	0	0	257.91	2.926	49.02	37.72	16	10	0	0
**V8**	-2.5	0	0	286.39	3.502	43.8	7.95	16	10	0	0
**V9**	-2.4	0	0	258.92	2.872	42.1	19.26	16	10	0	0
**V10**	-1.94	29.46	0.184	291.47	2.122	31.18	16.99	10	9	0	2
**V11**	-2.3	0	0	288.38	3.643	30.54	24.93	16	10	0	0
**V12**	-5.04	20.23	0.075	360.29	4.874	29.55	22.69	24	15	0	1
**V13**	-3.93	12.53	0.053	337.13	3.519	20.8	63.25	24	15	0	1
**V14**	-3.9	0	0	324.33	4.222	16.6	1.93	24	15	0	0
**V15**	-4.46	0	0	350.7	4.753	6.91	6.59	24	15	0	0
**V16**	-5.03	0	0	321.14	4.077	2.72	0.68	24	15	0	0
**V17**	-4.49	0	0	323.24	4.123	0.42	21.27	24	15	0	0
**V18**	-4.48	0	0	322.23	4.177	0.14	2.31	24	15	0	0

ID	Num_Atoms	Num_Bonds	Num_Hydrogens	Num_ExplicitHydrogens	Num_ExplicitAtoms	Num_ExplicitBonds	Num_PositiveAtoms	Num_NegativeAtoms
**V1**	17	19	13	0	17	19	0	0
**V2**	10	9	16	16	26	25	0	0
**V3**	10	11	16	16	26	27	0	0
**V4**	11	12	16	16	27	28	0	0
**V5**	10	11	16	16	26	27	0	0
**V6**	11	12	16	16	27	28	0	0
**V7**	10	11	16	16	26	27	0	0
**V8**	10	10	16	16	26	26	0	0
**V9**	10	11	16	16	26	27	0	0
**V10**	11	11	10	10	21	21	0	0
**V11**	10	10	16	16	26	26	0	0
**V12**	16	16	24	0	16	16	0	0
**V13**	16	18	24	24	40	42	0	0
**V14**	15	17	24	24	39	41	0	0
**V15**	15	16	24	24	39	40	0	0
**V16**	15	17	24	24	39	41	0	0
**V17**	15	17	24	24	39	41	0	0
**V18**	15	17	24	24	39	41	0	0

ID	Num_RingBonds	Num_RotatableBonds	Num_AromaticBonds	Num_BridgeBonds	Num_Rings	Num_AromaticRings	Num_RingAssemblies	Num_Rings3
**V1**	17	2	17	0	3	3	2	0
**V2**	0	4	0	0	0	0	0	0
**V3**	8	0	0	8	2	0	1	0
**V4**	8	0	0	8	2	0	1	0
**V5**	8	0	0	8	2	0	1	0
**V6**	8	0	0	8	2	0	1	0
**V7**	8	0	0	8	2	0	1	0
**V8**	6	1	0	0	1	0	1	0
**V9**	8	0	0	0	2	0	1	1
**V10**	6	2	6	0	1	1	1	0
**V11**	6	0	0	0	1	0	1	0
**V12**	6	2	6	0	1	1	1	0
**V13**	14	0	0	0	3	0	1	1
**V14**	12	1	0	12	3	0	1	0
**V15**	12	0	0	0	2	0	1	0
**V16**	13	0	0	8	3	0	1	0
**V17**	13	0	0	13	3	0	1	0
**V18**	13	0	0	13	3	0	1	0

ID	Num_Rings4	Num_Rings5	Num_Rings6	Num_Rings7	Num_Rings8	Num_Rings 9Plus	Num_Chains	Num_Fragments	Num_StereoAtoms	Num_StereoBonds
**V1**	0	0	3	0	0	0	1	1	0	0
**V2**	0	0	0	0	0	0	19	1	0	3
**V3**	1	0	1	0	0	0	19	1	3	1
**V4**	0	2	0	0	0	0	20	1	3	0
**V5**	1	0	1	0	0	0	19	1	3	1
**V6**	0	2	0	0	0	0	20	1	3	0
**V7**	0	2	0	0	0	0	19	1	3	1
**V8**	0	0	1	0	0	0	19	1	1	2
**V9**	0	0	1	0	0	0	19	1	3	1
**V10**	0	0	1	0	0	0	13	1	0	1
**V11**	0	0	1	0	0	0	19	1	0	2
**V12**	0	0	1	0	0	0	8	1	2	0
**V13**	1	0	0	0	0	1	28	1	5	1
**V14**	0	2	1	0	0	0	28	1	6	1
**V15**	1	0	0	0	0	1	28	1	3	2
**V16**	0	2	1	0	0	0	28	1	4	1
**V17**	1	0	1	1	0	0	28	1	5	1
**V18**	0	2	0	1	0	0	28	1	5	1

ID	Num_H_Acceptors	Num_H_Donors	Num_H_Acceptors_Lipinski	Num_H_Donors_Lipinski	HBA_Count	HBD_Count	NPlusO_Count	Organic_Count
**V1**	1	1	1	1	0	1	1	17
**V2**	0	0	0	0	0	0	0	10
**V3**	0	0	0	0	0	0	0	10
**V4**	1	0	1	0	1	0	1	11
**V5**	0	0	0	0	0	0	0	10
**V6**	1	0	1	0	1	0	1	11
**V7**	0	0	0	0	0	0	0	10
**V8**	0	0	0	0	0	0	0	10
**V9**	0	0	0	0	0	0	0	10
**V10**	2	1	2	1	1	1	2	11
**V11**	0	0	0	0	0	0	0	10
**V12**	1	1	1	1	0	1	1	16
**V13**	1	0	1	0	1	0	1	16
**V14**	0	0	0	0	0	0	0	15
**V15**	0	0	0	0	0	0	0	15
**V16**	0	0	0	0	0	0	0	15
**V17**	0	0	0	0	0	0	0	15
**V18**	0	0	0	0	0	0	0	15

Figure

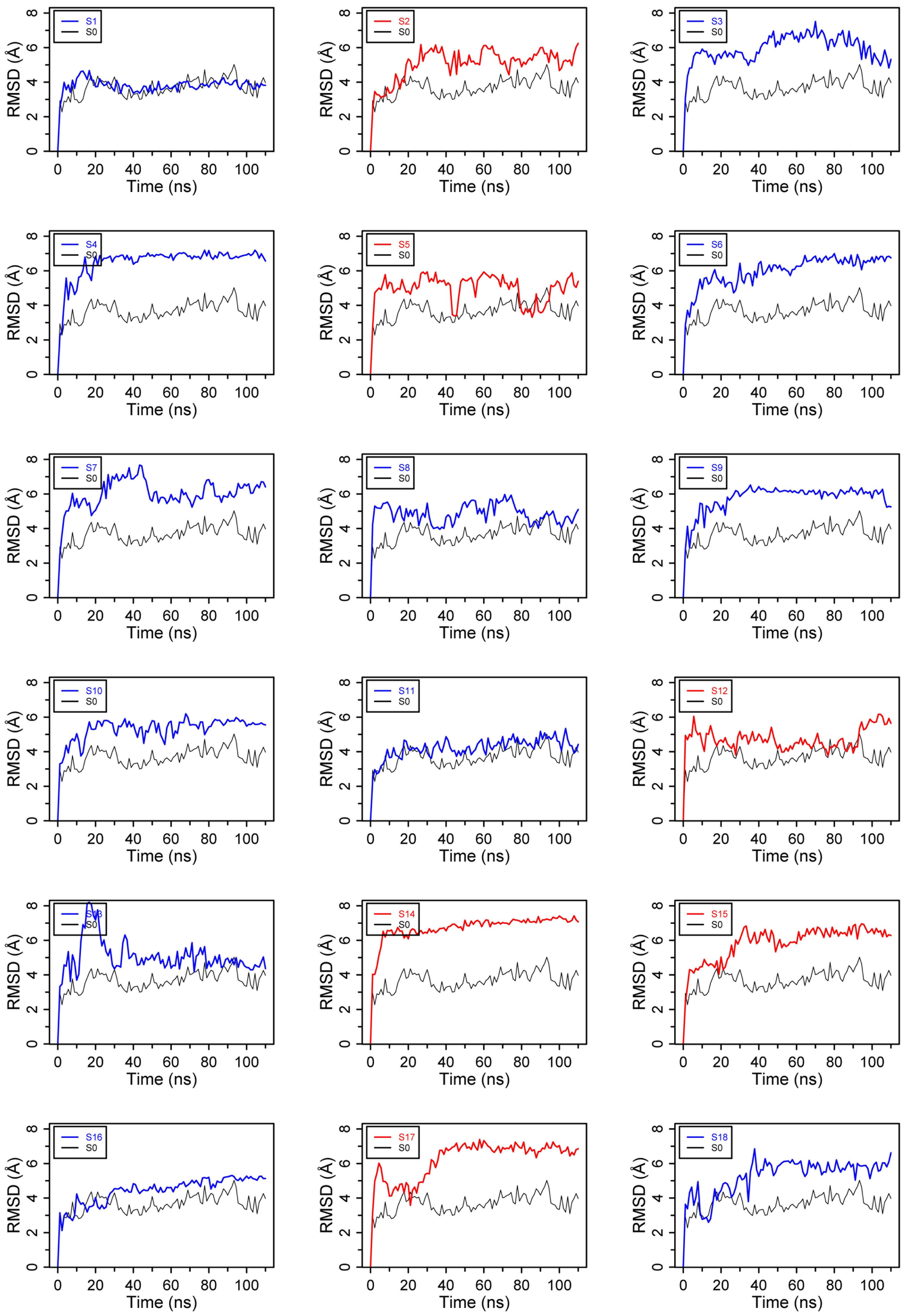

Figure S1. The RMSD of the protein main chain Cα of the 19 simulations. The RMSD of the six simulations (S2, S5, S12, S14, S15, S17) which underwent coil to helix transition were colored red, and the RMSD of S0 black, RMSD of the other 12 simulations blue.

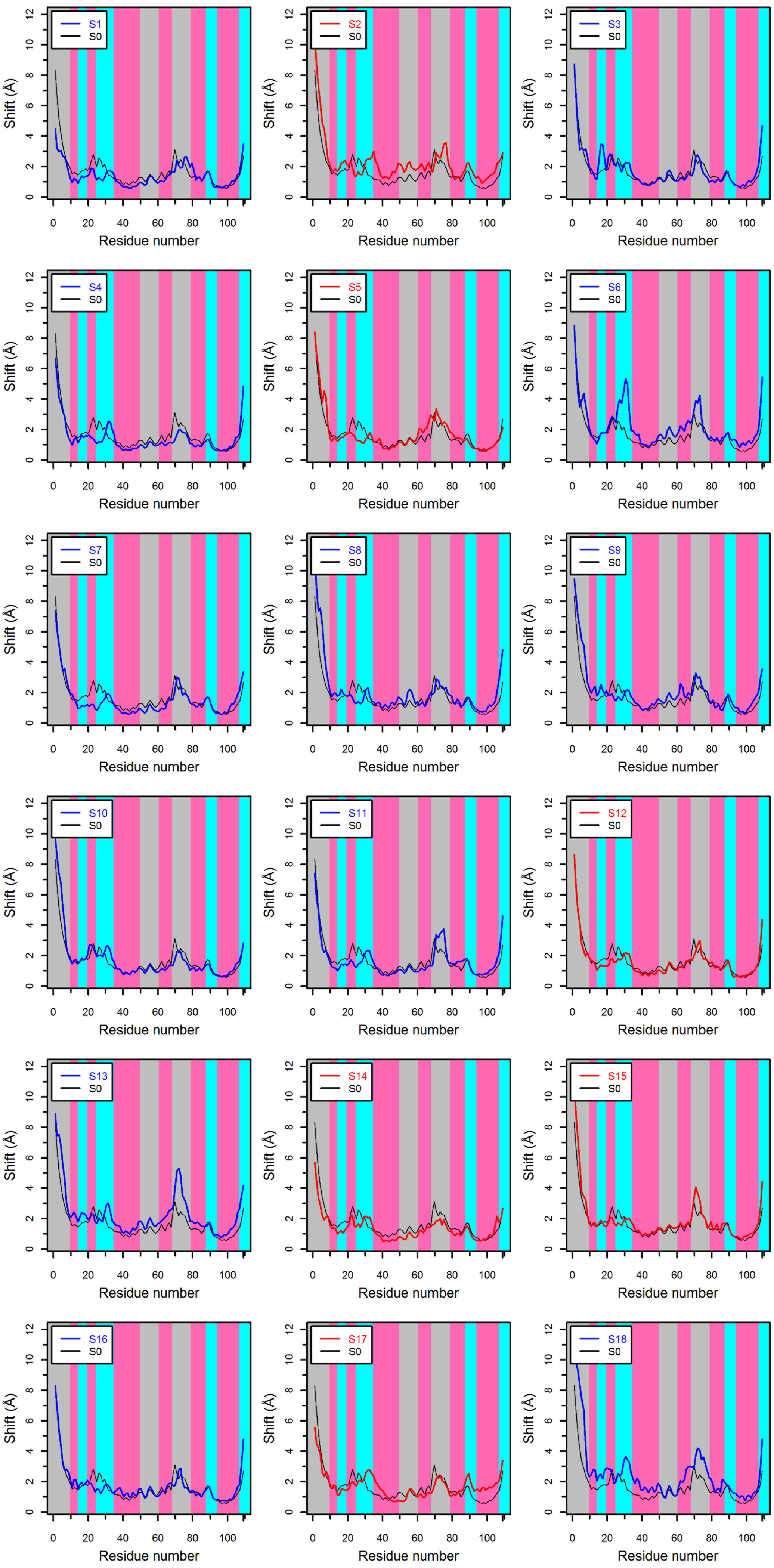

Figure S2. Average RMSF per residue of the protein backbone heavy atoms$_0$(Cα, N, C) of the 19 CMD simulations. The RMSD of the six simulations (S2, S5, S12, S14, S15, S17) which underwent coil to helix transition were colored red, and the RMSD of S0 black, RMSD of the other 12 simulations blue. The primary secondary structure of the protein's initial conformation was computed with the program DSSP and severed as the background for a better comprehension of the RMSF, which grey denoted random coil or bend, pink denoted alpha helix, blue denoted loop.

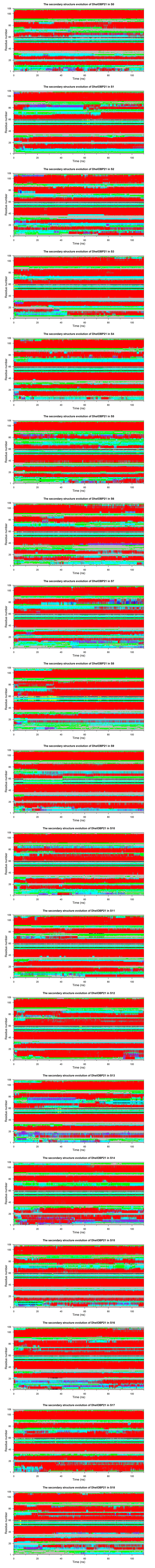

Figure S3. The secondary structural evolution of the DhelOBP21 in 19 CMD simulations. The 8 kinds of secondary structures were defined by program DSSP. RC refers to Random coil. And 6 systems (S2, S5, S12, S14, S15, and S17) have experienced an apparent structural transition which the random coil of the N-terminus gradually transformed into a regular alpha helix amongst the 19 systems.

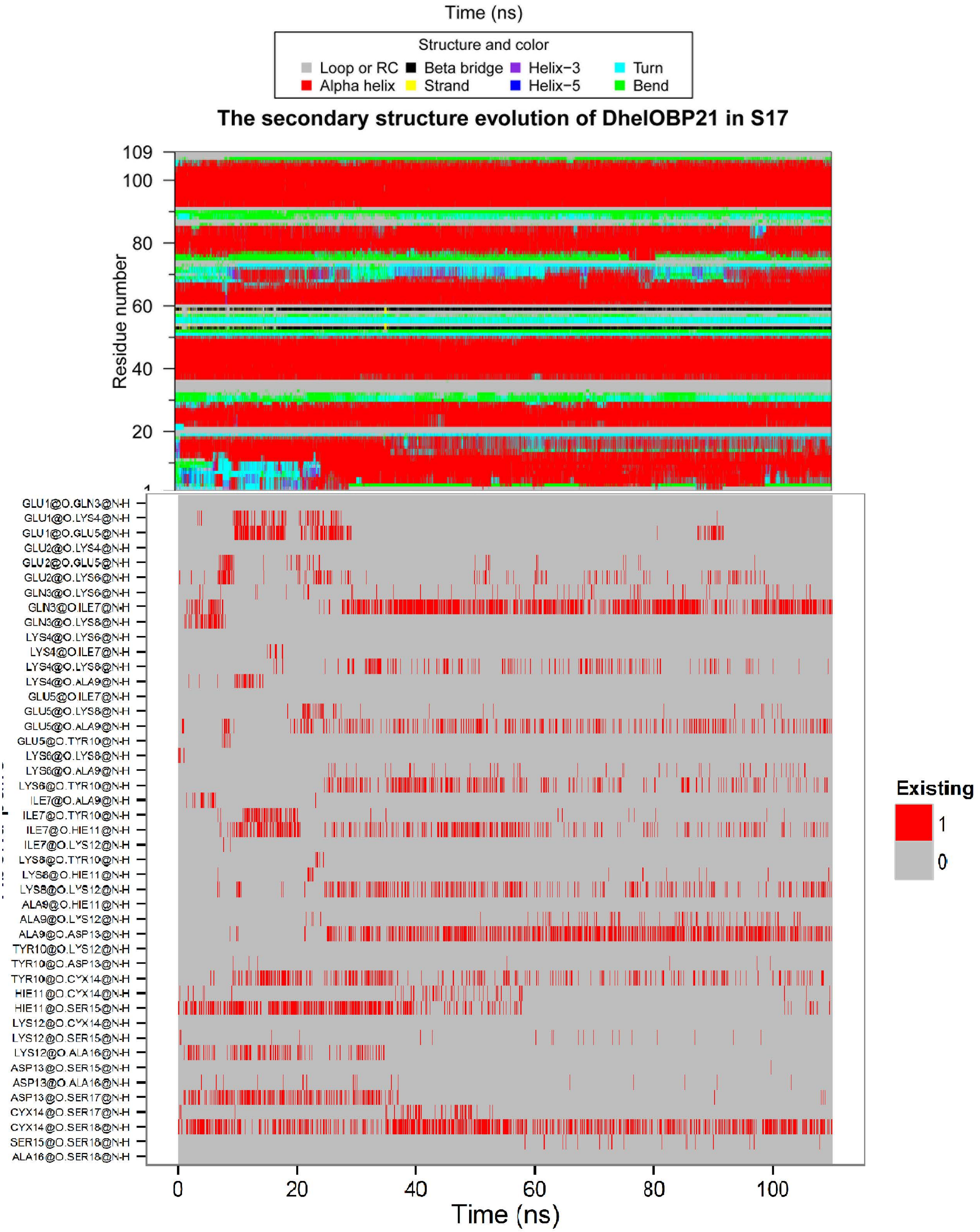

Figure S4. The hydrogen's evolution in the main chain of DhelOBP21 in S17 during the 110 ns CMD, where 1 refers to the existence of H-bond, and vice versa.

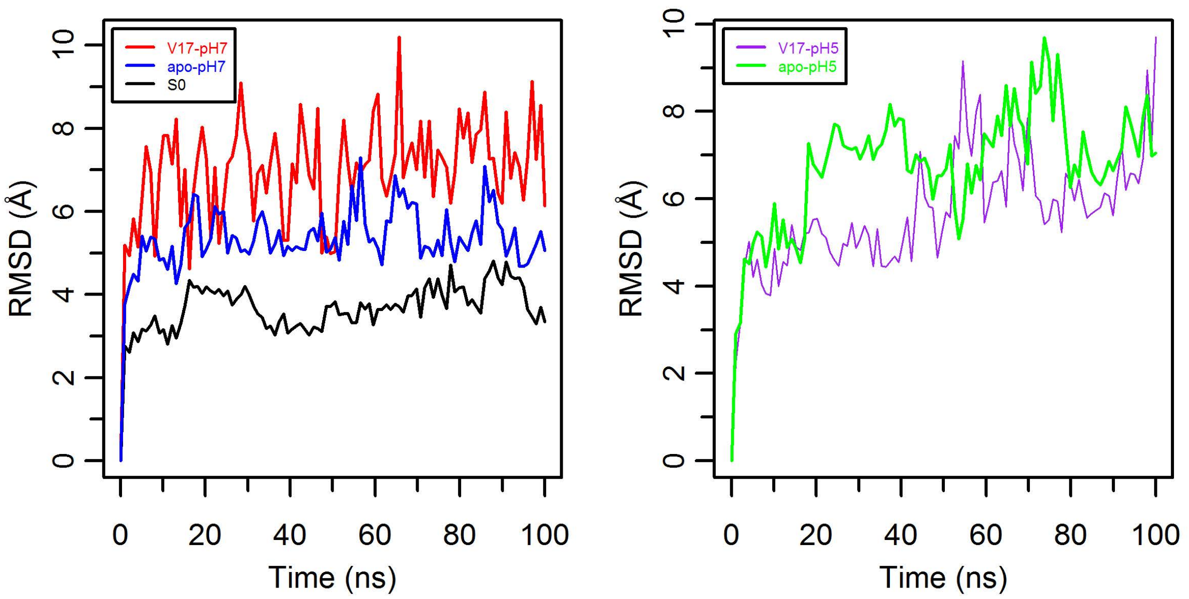

Figure S5. The RMSD of the protein main chain heavy atoms in the 4 CpHMD simulations. Note that the black line was the RMSD of the S0 (apo protein) in the CMD which was shown for comparison. While the red, blue, purple and green line refer to the RMSD of the holo state (with V17) protein (red) at pH 7.0, the apo state protein (blue) at pH 7.0, the holo state (with V17) protein (purple) at pH 5.0, the apo state protein (green) at pH 5.0.

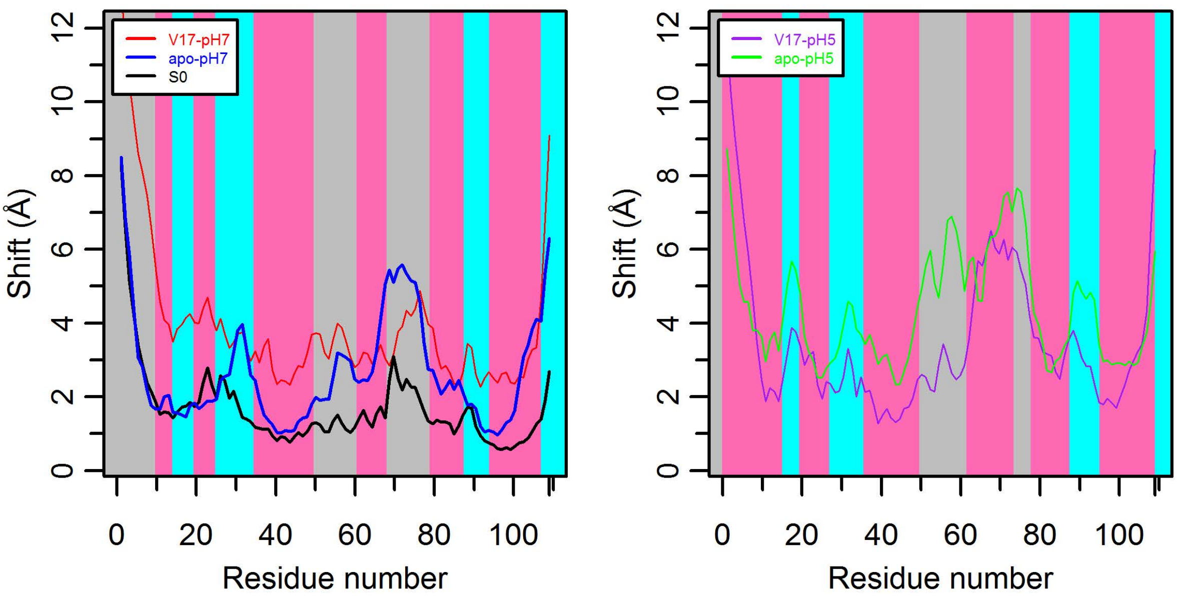

Figure S6. The RMSF of the protein main chain heavy atoms in the 4 CpHMD simulations. Note that the black line was the RMSF of the S0 (apo protein) in the CMD which was shown for comparison. While the red, blue, purple and green line refer to the RMSF of the holo state (with V17) protein (red) at pH 7.0, the apo state protein (blue) at pH 7.0, the holo state (with V17) protein (purple) at pH 5.0, the apo state protein (green) at pH 5.0, respectively.

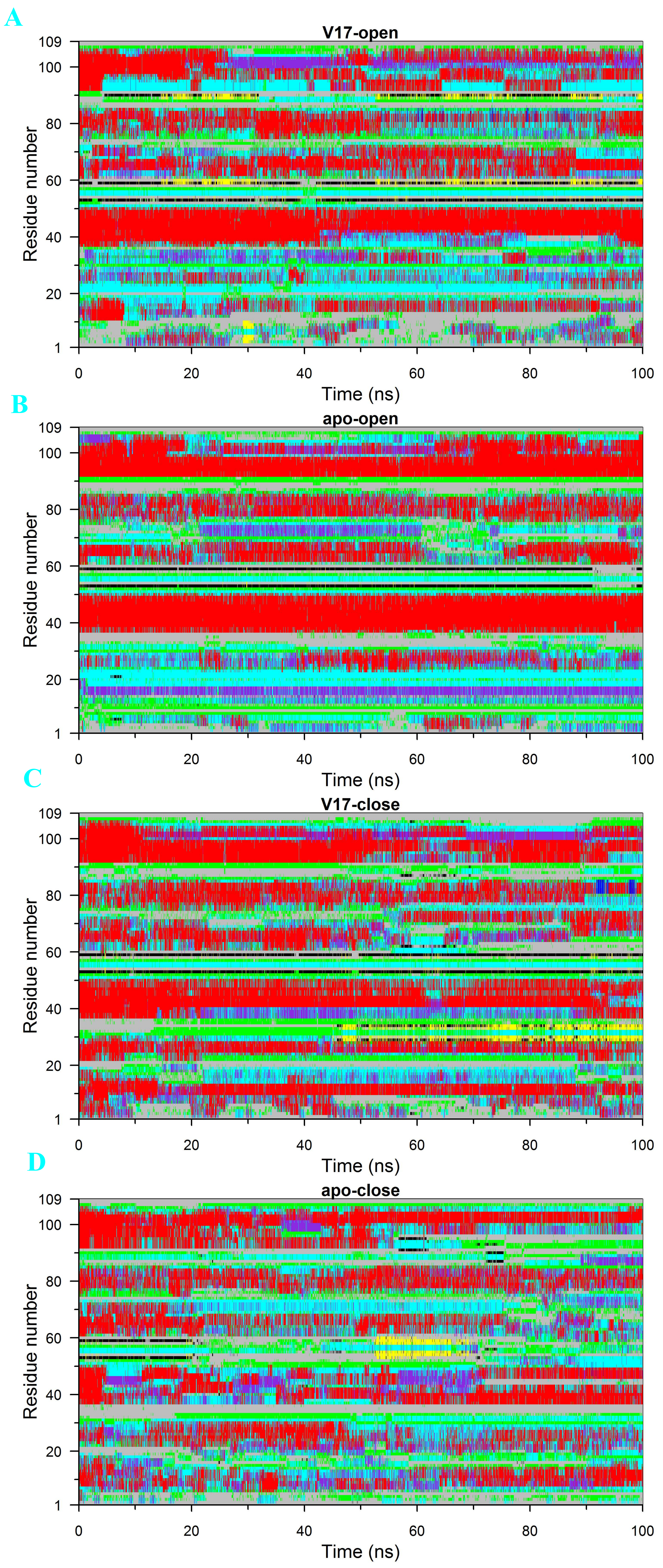

Figure S7. The secondary structure evolution of the protein in the 4 CpHMD simulations. the four figures refer to (A) the structural evolution of the holo state (with V17) protein at pH 7.0, (B) the apo state protein at pH 7.0, (C) the holo state (with V17) protein at pH 5.0, (D) the apo state protein at pH 5.0, respectively. V17 refers to (+)-Sativene.

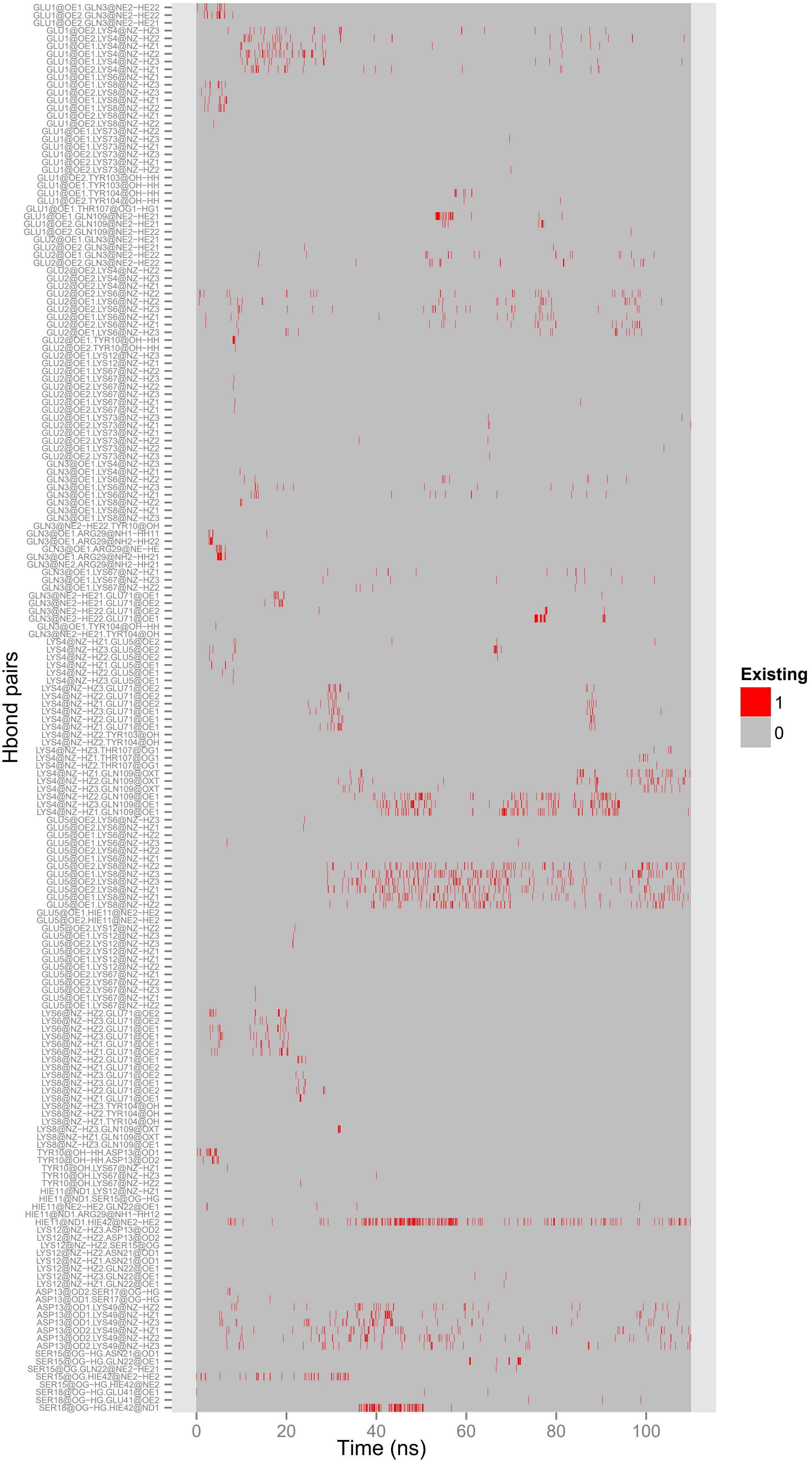

Figure S8. The main chain hydrogen bond evolution of the holo protein (with V17) with a closed pocket initially at pH 5.0.

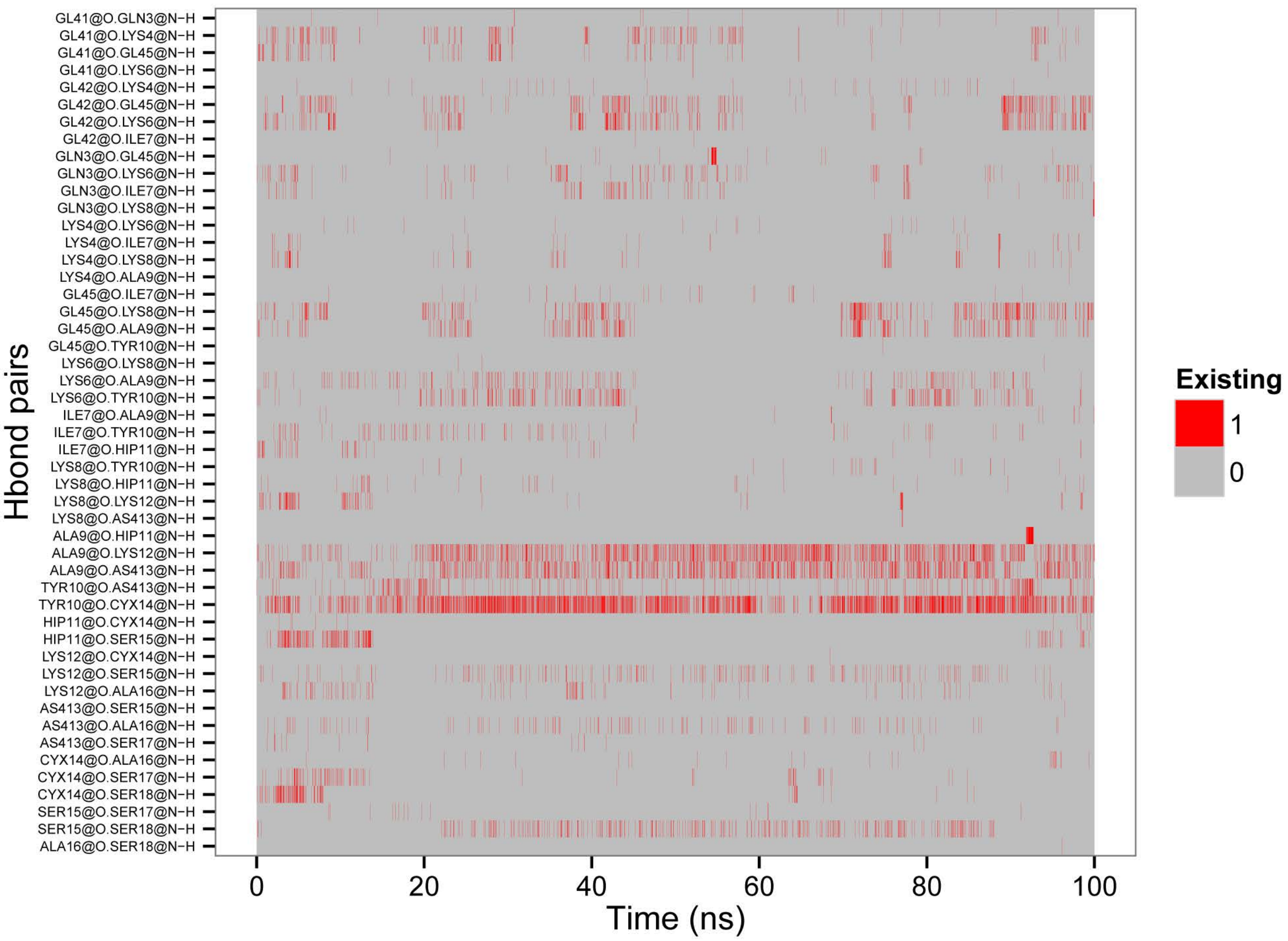

Figure S9. The sidechain hydrogen bond evolution of the holo protein (with V17) with a closed pocket initially at environmental pH 5.0, where 1 refers to the existence of H-bond, and vice versa.

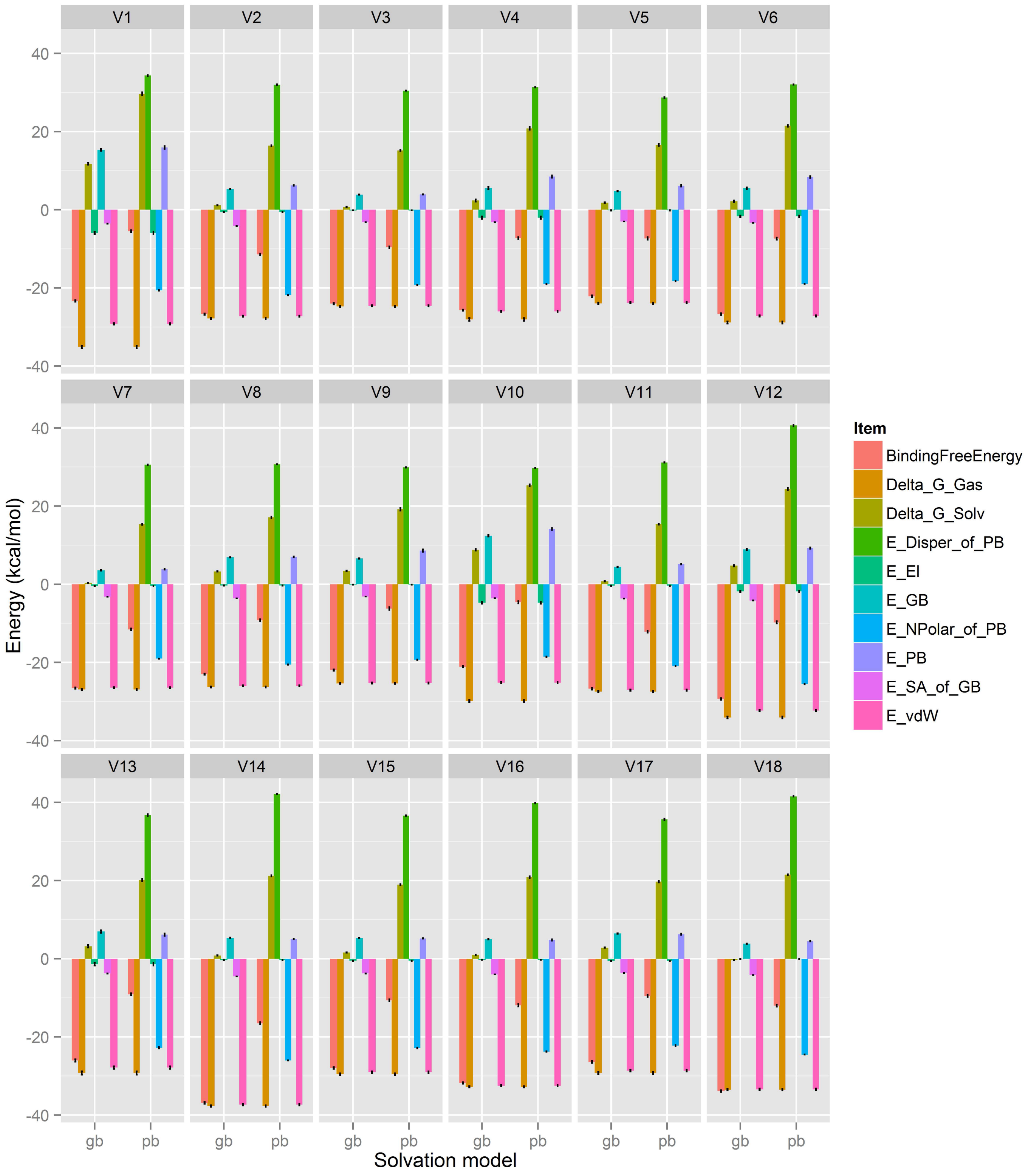

Figure S10. The decomposition of two kinds of binding free energy (MM-PBSA/GBSA). In the left (right) part of each subgraph were the MM-GBSA (MM-PBSA) decomposition items which were presented as bars 1 to 7 (8 to 15). In the left part (gb), the "BindingFreeEnergy" (bar 1, ΔGbind) consisted of Delta_G_Gas (bar 2, ΔGgas) and Delta_G_Solv (bar 3, ΔGsolvation). Besides, the "BindingFreeEnergy" (bar 1, ΔGbind) was the sum of E_El (bar 4, ΔEelectrostatic), E_GB (bar 5, ΔEGB), E_SA_of_GB (bar 6, ΔESA) and E_vdW (bar 7, ΔEvdw). By contrast, in the right part (pb), the "BindingFreeEnergy" (bar 8, ΔGbind) consisted of Delta_G_Gas (bar 9, ΔGgas) and Delta_G_Solv (bar 10, ΔGsolvation). Similarly, the "BindingFreeEnergy" (bar 8, ΔGbind) was the sum of E_Disper_of_PB (bar 11, one portion of ΔESA calculated in PB), E_El (bar 12, ΔEelectrostatic), E_NPolar_of_PB (bar 13, the other portion of ΔESA), E_PB (bar 14, ΔEPB), and E_vdW (bar 15, ΔEvdw).

Movie

Movie S1. The time evolving trajectory of the S17 for the 110ns (1100 frames). The V17 was rendered in green, and residues Arg29, Phe46, Phe52, Ile100 and Thr104 were colored yellow. Then the Glu1 and Gln109 represented the N- and C- termini, respectively.

Movie S2. The time evolving trajectory of the S17 for the first 10ns (1000 frames). The V17 was rendered in green, and residues Arg29, Phe46, Phe52, Ile100 and Thr104 were colored yellow. Then the Glu1 and Gln109 represented the N- and C- termini, respectively.

Movie S3. The time evolving trajectory of the S14 for the 110ns (1100 frames). The V17 was rendered in green, and residues Arg29, Phe46, Phe52, Ile100 and Thr104 were colored yellow. Then the Glu1 and Gln109 represented the N- and C- termini, respectively.

1. Li DZ, Yu GQ, Yi SC, Zhang Y, Kong DX, Wang MQ: **Structure-Based Analysis of the Ligand-Binding Mechanism for DhelOBP21, a C-minus Odorant Binding Protein, from *Dastarcus helophoroides* (Fairmaire; Coleoptera: Bothrideridae)**. *Int J Biol Sci* 2015, **11**(11):1281-1295.
